# Simulated dynamical transitions in a heterogeneous marmoset pFC cluster

**DOI:** 10.3389/fncom.2024.1398898

**Published:** 2024-05-28

**Authors:** Bernard A. Pailthorpe

**Affiliations:** Brain Dynamics Group, School of Physics, University of Sydney, Sydney, NSW, Australia

**Keywords:** marmoset, network, cluster, dynamics, transition, simulation

## Abstract

Network analysis of the marmoset cortical connectivity data indicates a significant 3D cluster in and around the pre-frontal cortex. A multi-node, heterogeneous neural mass model of this six-node cluster was constructed. Its parameters were informed by available experimental and simulation data so that each neural mass oscillated in a characteristic frequency band. Nodes were connected with directed, weighted links derived from the marmoset structural connectivity data. Heterogeneity arose from the different link weights and model parameters for each node. Stimulation of the cluster with an incident pulse train modulated in the standard frequency bands induced a variety of dynamical state transitions that lasted in the range of 5–10 s, suggestive of timescales relevant to short-term memory. A short gamma burst rapidly reset the beta-induced transition. The theta-induced transition state showed a spontaneous, delayed reset to the resting state. An additional, continuous gamma wave stimulus induced a new beating oscillatory state. Longer or repeated gamma bursts were phase-aligned with the beta oscillation, delivering increasing energy input and causing shorter transition times. The relevance of these results to working memory is yet to be established, but they suggest interesting opportunities.

## 1 Introduction

Marmoset provides a simpler primate brain (Solomon and Rosa, 2014) for both experimental and modeling studies. With a subdivided frontal lobe, it offers a valuable model system for exploring cortical functions. Network analysis (Pailthorpe, 2024) of the marmoset cortical connectivity data, measured by retrograde tracers (Majka et al., 2016, 2020), identified a significant 3D cluster in and around the pre-frontal cortex (pFC), which is examined here. The network analysis methods followed those used previously for the mouse retina (Pailthorpe, 2016) and the mouse brain (Pailthorpe, 2019). Previous studies of brain networks (Sporns and Kotter, 2004) generally focused on small motifs comprising 2, 3, or at most 4 nodes due to the combinatorial explosion in the number of possible configurations of larger clusters. Network analysis of the marmoset connectivity data (Pailthorpe, 2024) identified network hubs in or near the Marmoset pFC, visual cortex, auditory cortex, and somatosensory areas. These contained the leading hubs for in- or out-links along with a tight configuration of strong links to nearby nodes. The most striking is a closely coupled cluster of six anatomical areas in or near the Dorso lateral pFC (DlpFC), containing one out-hub (A10) and one in-hub (A32V), along with a connector node (A11), which is a marginal in-hub. Other participating nodes are A32, A9, and A46D.

The usual mesoscopic models of neural systems follow the original analysis of Wilson and Cowan (WC) and Jansen and Ritt (JR) (Wilson and Cowan, 1972, 1973; Jansen and Rit, 1995). A cortical column or anatomical area is modeled as a composite neural mass (NM), comprising three neural assemblies of excitatory or inhibitory neuronal populations. Studies typically employ a single NM or two identical interacting NMs, e.g., Jansen and Rit (1995). A number of prior studies form the background to the present work: David and Friston (2003) showed that a single NM generated a unimodal spectrum and further explored the influence of coupling strength and signal delays between two NMs; and David et al. (2005) explored the neuronal mechanisms underlying evoked response potentials resulting from impulsive stimuli or system parameter perturbations of multiple NMs. An extended model, incorporating fast and slow inhibitory subpopulations (Wendling et al., 2000), has been used to generate gamma-band oscillations; this model has been extended to three parallel NMs to model high-resolution EEG (Zavaglia et al., 2006) that exhibit multi-band spectra. Combined with an inverse method, this study located dipolar sources on the cortical surface at six areas of the human cortex: A46L & R, 5L & R, and areas 6AL & R, associated with somatosensory and motor functions. That approach focused on a different network hub region and complements the present work. Since one- and two-dimensional grids of identical NM (21; 16 × 16, 31 × 31 nodes) were interconnected by homogeneous but adjustable strength, nearest neighbor links were used (Goodfellow et al., 2012) to study propagating waves related to epilepsy as a function of coupling strength.

Here, those earlier studies are extended to multiple, interacting, unequal NMs. This study introduces three novel concepts: (1) it identifies an interesting six-node subsystem in the primate cortex using network theory applied to cortical structural links; (2) it introduces heterogeneous neural masses by tuning them to appropriate frequency bands; and (3) it introduces heterogeneous linkages between those neural masses using available directed, weighted connectivity data for marmoset cortex. Model outputs are the time-dependent local field potential (LFP) for each node and their average for the cluster. The complex overall outputs arise from constructive and destructive interference of the individual outputs from the linked oscillators. This six-node cluster exhibits a range of oscillations and phase plane behavior familiar from numerous earlier studies of neural masses, with added complexity due to the varying link weights, signal delays, and node heterogeneity.

The cluster exhibits sustained transitions in oscillatory behavior when stimulated by pulse trains modulated by oscillatory waveforms in the standard brain wave frequency bands (Buzsaki and Draguhn, 2004; Buzsaki, 2006). The output of A32V, the dominant in-hub, is the most responsive to the stimuli. Sustained dynamical transitions are observed, lasting up to 5–10 s. Gamma bursts can control the theta- or beta-induced transitions.

## 2 Methods

Marmoset cortex structural connectivity data, as measured by retrograde tracers (Majka et al., 2016, 2020) and available via a data portal (http://marmosetbrain.org), provide directed link weights between 116 anatomical areas based on 55 injection sites. That data are the basis of the present study, and its network properties have been analyzed (Pailthorpe, 2024) using a renormalization of the original fractional weight measure to reveal the underlying in-link weights. The network analyses of both datasets were compared, with the fractional weight measure predicting no in-hubs while still identifying A10 as an out-hub. The pFC cluster, with its two hubs, was only revealed by the inclusion of the in-link weights. The standard network techniques were applied to the renormalized data, including modular decomposition by InfoMap (Rosval and Bergstrom, 2007), motif classification (Sporns and Kotter, 2004), hub identification via participation coefficients (Guimerà and Nunes Amaral, 2005), temporal (time-evolving) network analysis (Holme and Saramarki, 2012), and tracing sensory pathways (Pailthorpe, 2019). That strategy of network analysis was arrived at in prior studies of simpler systems: the mouse retina (Pailthorpe, 2016) and mouse brain (Pailthorpe, 2019). Several modular decomposition algorithms were considered, with the InfoMap algorithm preferred since it is conceptually based on network traffic, i.e., multiple random walkers on the network, and has been shown to yield good results in a variety of networks (Fortunato, 2010). Limitations of these methods have been studied on a large collection of networks (Ghasemian et al., 2019) and reveal that InfoMap tends to over-fit the data, which provides good link description but poor link prediction. Neuron density and cortical thickness were reported by Atapour et al. (2019). The volume of each anatomical area was calculated by counting 3D voxels corresponding to each labeled area in the Marmoset brain atlas (Paxinos et al., 2012). Those quantities were required to calculate the number of neurons in each anatomical area. The calculated center of mass (centroid) of each area's voxels was taken to be the node coordinates and used to calculate inter-node distances.

### 2.1 Model and simulation equations

The WC/JR neural mass models have been widely studied from varying perspectives, as discussed above. Even so, it is worth reiterating that the differential equations in NM models have the form of a driven simple harmonic oscillator (Halliday et al., 1997):


(1) $$\begin{array}{l} \frac{d^{2}y}{dt^{2}}+k\;\frac{dy}{dt}+\;\frac{1}{\tau^{2}}\;y\left(t\right)=\;F_{ext}\left(t\right) \end{array}$$

Where *y*(*t*) is a time-dependent state variable (e.g., voltage), *k* is a damping coefficient that measures energy dissipation in the system, τ is the time constant characterizing system dynamics (e.g., decay) and *F*_ext_ is an external driving force. In an undamped system (*k* = 0), the oscillation frequency is ω = 1/τ. Non-linearities in such models enter via *F*_ext_. Equation (1) has been widely used and is well-characterized; in particular, the system's oscillation frequency is Halliday et al. (1997):


(2) $$\begin{array}{l} w={\left[\frac{1}{\tau^{2}}+{\langle \frac{k}{2}\rangle }^{2}\right]}^{\frac{1}{2}} \end{array}$$

When *k* = 2/τ, Equation (2) takes the form used in NM models, as in Equations (3)–(5). Then, ω = 0 and the system has no characteristic frequency and is a critically damped oscillator. Such a system is a “one shot oscillator” that quickly reverts to its initial state and thus does not sustain oscillations on its own—much like a well-tuned door closer. Its sustained activity only appears when driven by external inputs, such as external noise or stimuli, and by the outputs of linked nodes. Those inputs have a non-linear form, generally sigmoidal in shape, and generate non-linear dynamics. Overall, this is reminiscent of a neuron generating an action potential, followed by a refractory period. The neuron responds to external stimuli but, by itself, does not oscillate. Yet, collections of interacting neurons do. The origins of network oscillations in spiking (Wallace et al., 2011) and other systems have been reviewed (Brunel and Wang, 2003; Wang, 2010).

Casting the NM model in the form of Equation (1) provides intuition to guide the tuning of model parameters. The terms on the left are acceleration, damping force, and restoring force, while those on the right are the driving forces (stimuli and interactions). For stable oscillation, a reasonable balance of these terms is required; otherwise, the oscillation rapidly dies out, as it does for many parameter choices. Realizing the simplicity of Equation (1) invites the further analogy of coupled oscillators and the resulting frequency shifts caused by the coupling strengths. Extensions of Equations (1), (2) interacting oscillators have been well-studied for standard oscillator models, with explicit expressions for the resulting frequency shifts and amplitudes available (Jothimurugan et al., 2016). Such studies provide insight into tuning the frequency of each neural mass in the present study (cf. Supplementary material 2.5).

Each anatomical area in the cluster is a node modeled as three neural assemblies, or neural masses (NM), containing excitatory or inhibitory neurons, following WC (Wilson and Cowan, 1972, 1973) and JR (Jansen and Rit, 1995), originally formulated as models of a cortical column (Mountcastle, 1997; Molnar, 2013). The models used a measure of neuronal activity, such as the fraction of excited neurons or firing rate, and have also been interpreted using voltage, as adopted herein. The two views are equivalent given the monotonic relationship between the firing rate and voltage, noting that the signaling rate plateaus, or saturates, as voltage increases. The dynamics of each NM were originally described by two signal processing blocks that accumulate pulses to generate a post-synaptic potential and transform the voltage to a firing rate (Jansen and Rit, 1995). The resulting differential equation is just that of a critically damped linear oscillator. Its sustained activity only appears when driven by external inputs, from external noise or stimuli, and by the outputs of linked nodes. Those inputs have a non-linear form, generally sigmoidal in shape, and generate non-linear dynamics. The resulting coupled differential equations, when cast in the standard form (derivatives on the left and driving terms on the right), are as follows:


(3) $$\begin{array}{l} \frac{d^{2}y_{e}}{dt^{2}}+\frac{2}{\tau_{e}}\frac{dy_{e}}{d\;t}+\;\frac{1}{\tau_{e}^{2}}\;y_{e}\left(t\right)=\;\frac{A}{\tau_{e}}C_{2}\;S\left[C_{1}y_{0}\left(t\right)\right]\;\\ \;+\frac{A}{\tau_{e}}p\left(t\right)\;+\underset{j}{\sum }\;a_{kj}\frac{A}{\tau_{e}}S\left[dy_{j}\left(t\right)\right]\; \end{array}$$

(4) $$\begin{array}{l} \frac{d^{2}y_{i}}{dt^{2}}+\frac{2}{\tau_{i}}\frac{dy_{i}}{d\;t}+\;\frac{1}{\tau_{i}^{2}}\;y_{i}\left(t\right)=\;\frac{B}{\tau_{i}}C_{4}\;S\left[C_{3}y_{0}\left(t\right)\right] \end{array}$$

(5) $$\begin{array}{l} \frac{d^{2}y_{0}}{dt^{2}}+\frac{2}{\tau_{e}}\frac{dy_{0}}{d\;t}+\;\frac{1}{\tau_{e}^{2}}\;y_{0}\left(t\right)=\;\frac{A}{\tau_{e}}\;S\left[y_{e}\left(t\right)-\;y_{i}\left(t\right)\right] \end{array}$$

Where *y*_*e*_ and *y*_*i*_ are the potentials associated with the excitatory and inhibitory neural populations, respectively, *y*′ is its first-time derivative, and *y*" is the second derivative. Each equation is for a single node *k* (= 1–6), and the sum in Equation (3) is over linked nodes *j*. *y*_0_ is the potential of the subpopulation of excitatory neurons that are driven by the difference *dy* = *y*_*e*_ – y_i_. The time constants τ_*e*_ and τ_*i*_ (~ 10 ms) represent the joint effects of “delays associated with the membrane resistance and distributed delays in the dendritic network” (Jansen and Rit, 1995) in the excitatory and inhibitory neural populations. The units throughout Equations (1)–(3) are V/s^2^, although mV/s^2^ is used in practice. The presence on the left of coefficients 2/τ_*e*_ and 1/τ^2^_*e*_ indicates that the oscillator is critically damped, as discussed above. The terms on the right are the non-linear feedback and driving inputs; the latter are applied only to the excitatory population. These internal and external driving forces are what cause the system to produce sustained oscillations, overcoming the critical damping. Casting the model in the form of Equations (1), (3)–(5) aids in the tuning of the parameters. It also makes clear the balance of driving forces, particularly between the excitatory and inhibitory sub-populations.

The input p(t) is the background noise due to local cortical activity. The final term in Equation (1) is the summed inputs to the current node (*k*) from linked nodes (*j*), weighted by the adjacency, or connectivity, matrix elements *a*_*kj*_. Numerous authors have discussed the adjacency matrix and its use in neuroscience; that literature is best captured by reviews by Bullmore and Sporns (2009) and Bassett and Sporns (2017) and textbooks by Sporns (2011). In Equation (3), *dy*_*j*_ = *y*_*e*_ – *y*_*i*_ is the output of each linked node j (in mV) and is the difference in voltage outputs of the excitatory and inhibitory neural subpopulations that are presented to the pyramidal subpopulation in each node. Following Jansen and Rit (1995) and Goodfellow et al. (2012), the model output, *dy*_*j*_, is taken to be a local field potential for that anatomical area. LFP for the cluster is taken as the average of the six contributions. Signal delays can be introduced in this term by using instead a time-delayed *dy*_*j*_ (*t* – *d*_*ij*_/*v*), where *d*_*ij*_ is the inter-node distance, available from atlas coordinates, and here *v* is the local signal velocity. Local signals were assumed to travel along axonal or other pathways at 1 m/s (Muller et al., 2014), assuming short-range links may be unmyelinated. The coefficients A and B, originally the maximum post-synaptic potential amplitude, also quantify the feed-forward synaptic strengths, while C_1_-C_4_ describe the feedback strengths to the excitatory and inhibitory populations (cf. Supplementary material 2.2). The function S (units Hz or s^−1^) is the voltage to firing rate transformation, described below. Parameter values are discussed below and are taken to be broadly consistent with those used in previous studies (Wilson and Cowan, 1972, 1973; Jansen and Rit, 1995; David and Friston, 2003). Derivation of the average synaptic weights *w*_av_ from available data is discussed below. Note that there can be a separate *w*_av_ for in and out links, so the function S may vary.

### 2.2 Numerical methods

These equations were solved numerically by a 4th order Runga Kutta method (Press et al., 2007), with a timestep of 0.1 ms, using MATLAB codes. Recasting each 2nd order differential equation as two 1st order equations improves numerical stability. Small background noise (Gaussian random) was applied; alternatively, the use of uniform or no noise did not affect the results. Signal delays mean that Equations (3)–(5) become delay differential equations (cf. MATLAB Help Menu). They are of a simple type since delays only appear in the driving terms on the rights of Equations (3)–(5), so they are straightforward to solve. Transients died out within 2–3 s of simulation; stimuli were initiated at 4 s, and the simulation ran for a total of 20 s. Longer duration responses were simulated out to 30 or 40 s as a check. Fast Fourier Transforms of model outputs (voltage *dy*), using the MATLAB Signal Processing Toolbox, were used to find the spectral density distribution of each NM's output to identify prominent spectral peaks (MATLAB *findpeaks* function) and to quantify the distribution of power across frequency bands.

### 2.3 Voltage transformation to signaling rate

The voltage-to-rate transformation function S is usually approximated by the convenient sigmoid function, which can be derived from the distribution over voltage thresholds for firing in a neural population of fixed synaptic weight (Wilson and Cowan, 1972). However, measured firing thresholds vary by only 10s% (Henze and Buszki, 2001; Yu et al., 2008), while connectivity data of link number and link weights can vary by 2–3 orders of magnitude, as with the present data. In that case, a distribution of synaptic weights in a neural population with the same threshold, v_0_, is appropriate, as originally noted (Wilson and Cowan, 1972). That derivation (Supplementary material 2.1) yields a new transformation function:


(6) $$\begin{array}{l} S\left(v\left(t\right)\right)=q_{\text{m}}\;erfc\;\left(-r\left(\frac{v_{0}}{v\left(t\right)}-\;w_{av}\right)\right) \end{array}$$

Where *q*_*m*_ is the maximum rate, *v*_0_ is the mid-point (50% firing rate) of the sigmoid curve, *w*_av_ is the average synaptic weight in each neural population, and *r* is the inverse of its standard deviation, and also the steepness of the sigmoid. The function erfc is the complementary error function (Arfken, 1970).^1^ This is a steeper curve than the usual sigmoidal form (cf. Supplementary Figure S2).

### 2.4 Heterogeneous nodes

Many simulations use identical neural masses, in which model parameters are identical for each node. Neural masses are used to model cortical areas or columns, so it is evident from anatomical and other observations that the neural masses need to differ. That can be captured in the internal model parameters described above. An evident variation between anatomical areas is in volume V and number N of neurons in each area, followed by the varying number and weights of the in- and out-links, here captured by *w*_av_. All of those are available from the marmoset data used here. The internal time constants, τ_*e*_ and τ_*i*_, are not known but generally fall in the range of 10–25 ms in keeping with known neural time constants (Koch et al., 1996). The values of *N*, *w*_av − in_, and *w*_av − out_ vary significantly across the six nodes of the cluster studied here, so it is likely that the emergent dynamics of those nodes may vary.

#### 2.4.1 Link weights

The rich detail in the marmoset connectivity data facilitated the estimation of the parameters of the synaptic weight distribution w_av_ and r (reciprocal of the standard deviation). Details are presented in Supplementary material 2.4. The cluster is compact and almost fully linked, compared to the sparse linkage of the cortex overall (26%). Links of the six areas, along with all other areas of the cortex, comprise 15% of all links detected in the cortex. A summary of link weight data internal to the cluster is presented in Supplementary Tables S1, S2, and external link data are summarized in Supplementary Table S3.

#### 2.4.2 LIF model of an NM informs parameter choice

A Leaky Integrate and Fire (LIF) model of an NM (Mazzoni et al., 2008, 2011) has been used to characterize LFP. Here, that model was also used to study the dynamics and characteristic frequencies as a function of each NM's internal parameters and size. Details are presented in Supplementary material 2.6. LIF simulations showed f increasing with *N* up to *N* ~ 400 neurons and then constant or decreasing slowly with larger *N*, out to *N* =10,000 (Supplementary Figure S5). For comparison, simulations for the six nodes also indicate that oscillation frequency increases linearly with size *N* (Table 1; Supplementary Table S2). Geometric considerations of the marmoset anatomical areas (Supplementary Table S7) show consistency with these simulations in that f increases with *N* for both. Thus, the LIF model also provided guidance in tuning frequencies for the 6 NMs used to model the pFC cluster.

**Table 1:** Parameter values (Equations 3–6) and resultant frequency bands were found for the six nodes in the pFC cluster.

**Node**	**Frequency band**	***C***	***r***	***w*_av_**	***τ_*e*_* (ms)**	***τ_*i*_* (ms)**	***f* (Hz)**
A10	Gamma	220	0.5	3	5	5	32.1
A32V	Theta	200	0.5	5	25	25	5.3, 10.7
A32	Beta	250	0.5	3	10	10	16.0
A9	Alpha	220	0.5	1.5	16	17	9.9
A46D	Theta	200	0.5	3.5	25	25	5.9
A11	Beta	250	0.5	3	10	10	16.1

Reported *w*_av_ was the optimum found amongst 10 trials. The dominant frequency f was found from peaks in the power spectral density of LFP for each node; secondary peaks are listed when significant.

#### 2.4.3 Frequency band assignment

Little appears to be known about the natural frequency bands associated with NM models of individual anatomical areas. Presently, multi-electrode electrode arrays (Fukushima et al., 2014), which have been used in the marmoset parietal cortex (Komatsu et al., 2015), may yield LFP recording at sufficient spatial resolution to shed light on that question. Here, frequency band assignment to the 6 nodes emerged from exploratory simulations of 1, 2, and 4 node clusters and the inclusion of link weight data (Supplementary Tables S1, S2), along with the LIF simulations of NM models of varying size. Exploratory simulations, described in Supplementary material 2.5, provided indicative value of the parameters, listed in Table 1 and Supplementary Tables S4, S5. The familiar theta (4–8 Hz), alpha (8–12 Hz), beta (12–30 Hz), and gamma (30–100 Hz) bands consistently appeared during parameter searches, or no oscillation occurred. This suggests that these bands are a natural feature of the WC/JR model of an NM. Together with the LIF simulations, these searches suggested a possible assignment of frequency bands amongst the six nodes. Some combinations of parameters were tested, with about 10 producing viable oscillations in all six nodes. Comparisons of that simulation w_av_ with weight/link and weight/neuron data for marmoset (Supplementary Figures S3, S4) suggest the latter is a better measure of synaptic weight in Equation (6). The optimal assignment used in subsequent simulations is listed in Table 1.

It was found that r was the least sensitive parameter and that *r* = 0.5 sufficed; for simplicity, the constant value was used throughout. Note that lower *r* shifted spectral power to the alpha and beta bands. A32V had dominant (~80%) theta power but also had spectral power (10%) in the alpha and beta bands and sometimes shifted between theta and beta bands depending on parameters and stimuli. Possibly, this was due to its strong in-links from A10 and A9 and sensitivity to their dynamics. Overall, nodes showed no preference for a frequency band, with the emergent frequencies generally following the parameter assignment. Typical parameters for each frequency band are presented in Supplementary Table S1. Other illustrative calculations are summarized in Supplementary Table S5.

### 2.5 Stimulus

Random noise (zero mean and unit variance) was used to model inputs to an NM from surrounding neural regions, as is usually used. It is also known that wave-like oscillations propagate across the brain, some associated with cognitive tasks (Bhattacharya et al., 2022). Neural field theory provides a detailed description of electromagnetic waves in the brain that manifest as standing waves, or eigenmodes (Robinson et al., 2016), and traveling waves (Gabay et al., 2018). Waves communicating between distant brain regions have been observed in marmoset (Davis et al., 2019). Propagating beta band LFP waves have been shown to facilitate information transfer in motor cortical areas of macaque (Rubino et al., 2007) and to be present in the human motor cortex (Takahashi et al., 2011). Theta and alpha traveling waves have also been observed in the human neocortex, correlated with a memory task (Zhang et al., 2018). In view of this and other evidence, simulated wave-like stimuli were applied to the pFC cluster.

To simulate a wave like stimulus, a signal comprising a constant pulse rate, modulated by a sin wave in one of the four bands, was constructed. The stimulus, being a pulse rate, must be strictly positive (cf. Supplementary Figure S9). The options to achieve this are explained in Supplementary material 2.9. The model was stimulated by pulse train waveforms with amplitude modulated by an oscillatory waveform in each of the theta to gamma bands. The frequency was chosen to match the spectral peaks in the resting state simulation (cf. Table 1). The stimulus was turned on at 4.0 s, well-after transients had settled down, and generally applied either for the full simulation run (20 s). When present, a second stimulus was applied at 10 s. Stimuli were applied to all nodes or to nodes singly, in pairs, etc. When needed, simulation runs were extended to 30 or 40 s if further responses persisted. A pulse function stimulus was also constructed following the original methods (Jansen et al., 1993), using a pulse density function of a monophasic exponential form: *t*^7^exp(–*t*/*t*), with a 4-ms time constant. Its maximum amplitude of 200 Hz matched that of the wave stimulus and delivered 5.2 “unit pulses” (unitless: Hz ^*^ s) as a proxy measure of energy input. For comparison, a 16-s wave-modulated stimulus typically delivers ~1,000 unit pulses over the duration of the stimulus.

## 3 Results

The basic network analysis, modular decomposition, and hub classification of the marmoset cortical connectivity data have been presented (Pailthorpe, 2024). Modular decomposition by the InfoMap method (Rosval and Bergstrom, 2007) yielded eight modules clustered around hub or connector nodes and is broadly confirmed by the Louvain method (Blondel et al., 2008). The module associated with pFC was the third in significance as measured by probability flow. Motif analysis (Sporns and Kotter, 2004) showed a widespread prevalence of triangles with motif #8, a counterclockwise triangle comprising 51% of the triangles in pFC. Motif analysis alone would not have identified the six-node cluster studied herein; which was only found after detailed network searches, particularly time-evolving network tracing and visualization.

Through this, hubs in pFC, sensory areas, and association areas were identified, with the pFC hubs being the most prominent. The six-node cluster studied here comprises areas A10, A32V, A11, A32, A9, and A46D. Three of the nodes are in DlpFC, and three are in the nearby orbital or medial lobes. Some 60% of the total weight of all cluster links is internal to the cluster, indicating the prevalence of internal links. Separately, parcellation-free, column scale connectivity data, including also anterograde tracers, for marmoset has become available (Watakabe et al., 2023) but has not yet been analyzed.

**Figure 1:**
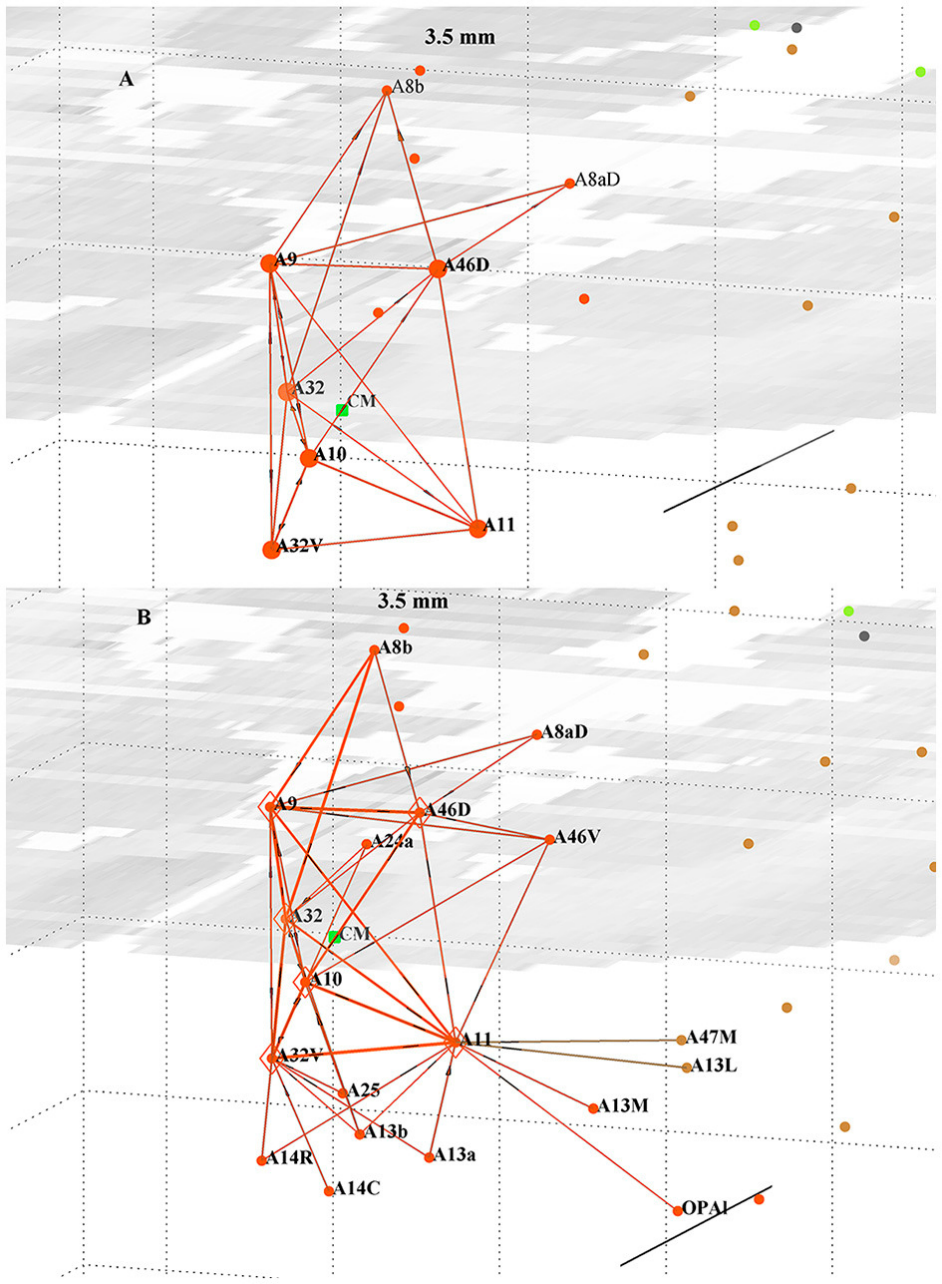
Perspective view of the six-node cluster around pFC in marmoset, forming an approximate square pyramid in 3D; **(A) (top)** showing all out-links and **(B) (bottom)** all in-links within 3.5 mm of any of the cluster nodes. The 13 external nodes are within the size scale of the cluster. View from the anterior, left side. Stronger links are shown by thicker lines. Centroid of the cluster is denoted by the green dot. The mid-horizontal plane image is a slice from the marmoset cortex volume image (Paxinos et al., 2012; http://marmosetbrain.org) to aid perspective; that volume image has 0.04 × 0.04 × 0.5 mm sized voxels, as evident in the image. The background grid is 2 mm squares and the A-P scale bar is 5 mm, here seen in perspective view. Nodes and links are color coded by Infomap module membership (Pailthorpe, 2024), as listed in Table 1.

The six-node cluster is geometrically pyramidal shape in three-dimensional (3D) space, centered around the dominant out-hub A10, and is topologically compact, as shown in Figure 1. The other five nodes are within 3.4 mm of the central node A10 and, with an assumed local conduction velocity of 1 m/s, can signal within 3.4 ms. The two next nearest nodes (A8aD and A8b) are within 5 mm and have only five weaker links with the core cluster, along with the 13 next closet nodes (each within 3.4 mm of any cluster node) that have only one or two links each to the cluster. All 116 nodes are plotted and color coded according to Table 1. Only links with the cluster are plotted. The distance scale is indicated by the background grid and the scale bar (cf. Caption). The cluster is an almost fully connected sub-network. The internal links are quite strong: one-third have weight >1,000 and are amongst the strongest links in the cortex, and another quarter have weight >500. The next closest node with a link weight >78 (i.e., top 40% of links) is at 11.3 mm, with a few lower-weight links present. Together, these features suggest a soft boundary and that the six nodes form a structurally distinct cluster. A model of an eight-node cluster, including areas A8aD and A8b, was also studied (not reported) and exhibited generally similar behavior. The six-node cluster and its nearest in- and out-links are shown in Figure 1, with internal link distances < 3.5 mm. The six nodes are more densely interconnected and have 93% of the total link weight present in the figure, with the balance to the additional 13 nodes nearby, as shown in Figure 1. The maximum link distance, 3.4 mm, is taken as a distance scale to characterize the cluster. The set of neighboring nodes within that distance was also explored to better understand the boundary of the cluster, which necessarily is poorly defined. Those 13 external nodes have weaker in-links (Figure 1B) to the cluster and few out-links (Figure 1A).

### 3.1 Comparison of models

The original NM model, which uses the sigmoid voltage-rate transformation function (Supplementary Equation S2), produces LFP outputs that are combinations of simple sinusoidal functions, as illustrated in Supplementary Figure S5. Some heterogeneity is available via the nodes' internal parameters. Furthermore, mild heterogeneity can be introduced by modifying the sigmoid to include average synaptic weights, *w*_av_ (Supplementary Equation S3), yielding similar results. These simple models did not produce dynamic transitions in response to stimuli. The extended NM model, used herein, uses integration over a normal distribution of synaptic weights to yield a steeper, shifted voltage-rate transformation function (Equation 6; Supplementary Equation S4; Supplementary Figure S2) that is more sensitive to w_av_ and is the focus of the present study. This *w*_av_ can be estimated from available linkage data for the marmoset cortex (Supplementary material 2.4; Supplementary Tables S1, S2). Searching parameter combinations produced both simple and complex dynamics. Two (#2, #6 out of 10) trials yielded sustained transitions in the dynamical state following wave-like stimuli of selected nodes in the cluster. The results presented here are for a single set of parameters (trial #6) using the parameters listed in Table 1. A typical output of the model in a resting state, driven only by noise, is shown in Supplementary Figure S7. The LFP for the cluster has spectral power distribution: 63% theta, 14% alpha, 21% beta, and 1.7% gamma bands. A representative Fourier spectrum is shown in Supplementary Figure S8 and is compared to available data, which shows strong beta peaks.

### 3.2 Response to wave stimuli

**Figure 2:**
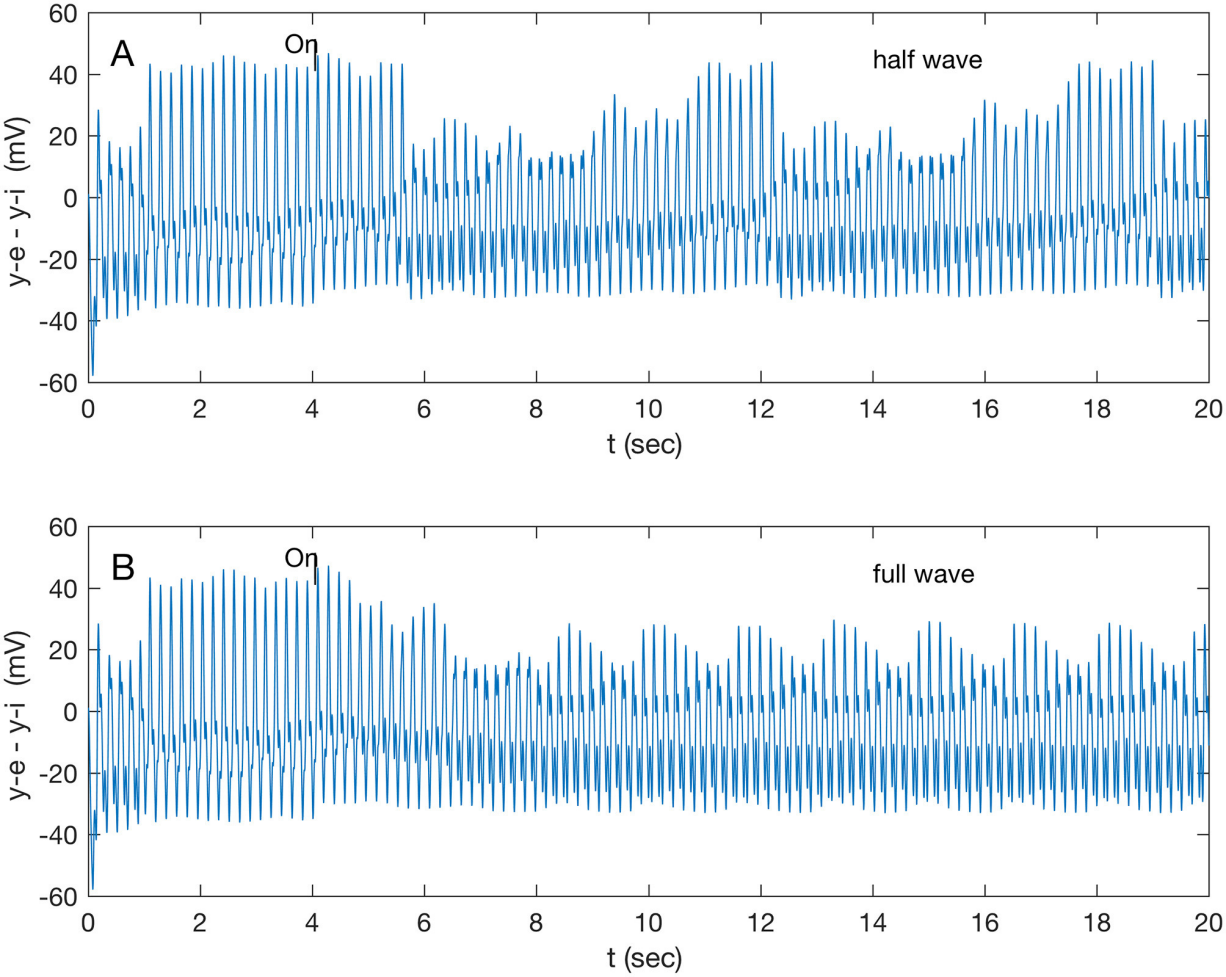
Response of node 2 (A32V) to a gamma band (32.1 Hz) modulated 200 Hz stimulus applied to both nodes 2 and 6 (A32V and A11) at 4 s; **(A)** response to half-wave stimulus, and **(B)** response to full-wave stimulus.

Stimuli were applied to all nodes and selectively to single and pairs of nodes. Node 2 (A32V) showed a distinctive response, presented below, while the other target nodes showed a small (~2 mV) step up in potential with an unchanged waveform. Other nodes, not targeted, showed no response. A gamma (32.1 Hz) stimulus to both A32V and A11 caused the responses shown in Figure 2. The biased full wave and the half wave stimulus caused identical responses, with varying delays in onset. The half wave stimulus (Supplementary Figure S9B) induced a complex, ramped, beating waveform after a 1.6-s transition (Figure 2A), repeating after 6.6 s; while the full wave stimulus induced a simple beat (Figure 2B) after a 2.6 s transition. Note that the full wave stimulus (Supplementary Figure S9A) is effectively oscillating at twice the input frequency (i.e., 64.2 Hz) while still in the gamma band. A check, by applying a double frequency (64.2 Hz) half wave stimulus to both A32V and A11, confirmed a response (not shown) similar to that presented in Figure 2B, but with a longer repeat time of 8.6 s.

**Figure 3:**
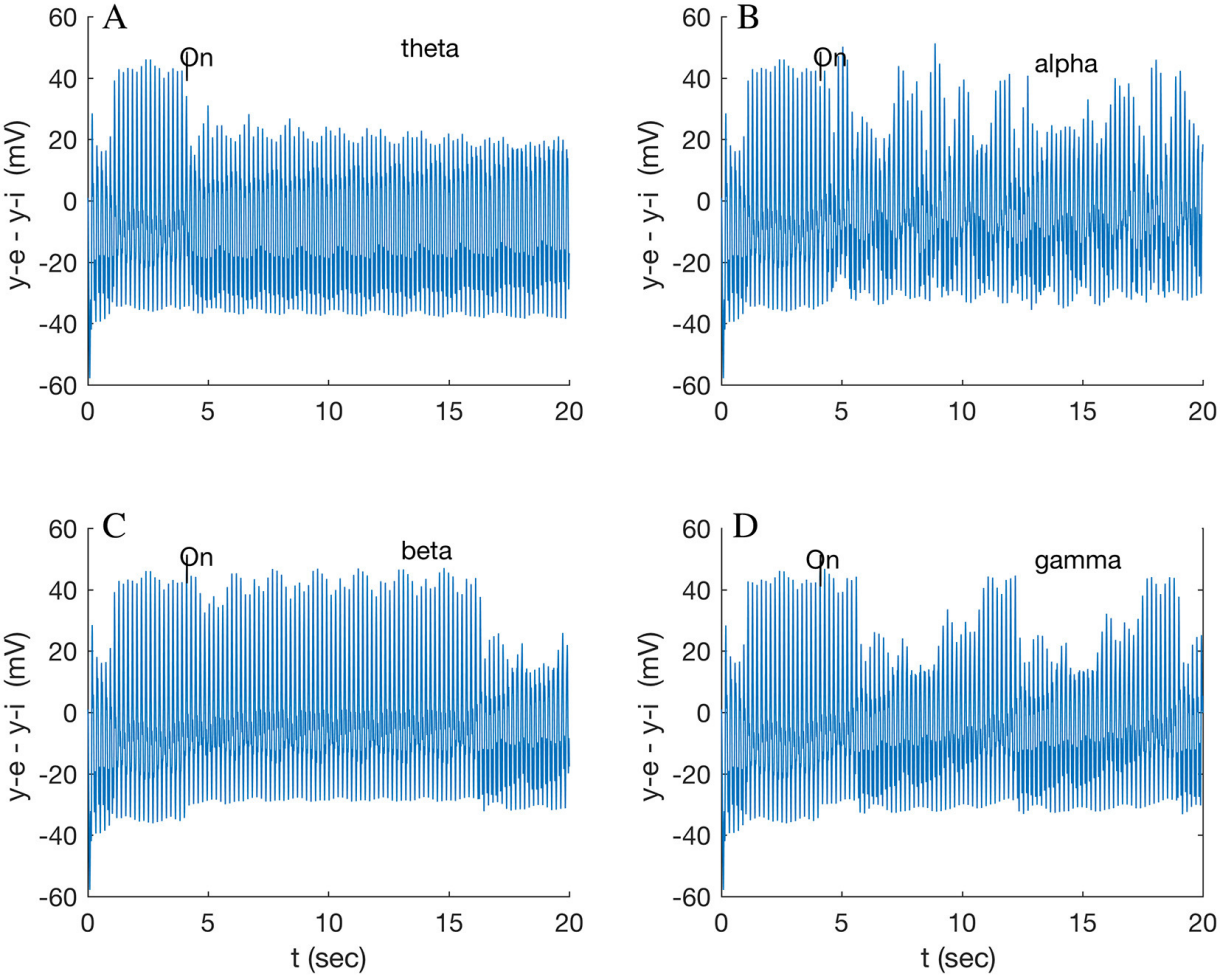
Response of node 2 (A32V) to a 200 Hz stimulus modulated by each of **(A)** theta (5.3 Hz); **(B)** alpha (9.9 Hz); **(C)** beta (16.0 Hz); and **(D)** gamma band (32.1 Hz). Stimuli applied to both nodes 2 (A32V) and 6 (A11) at 4 s.

A full-wave gamma stimulus applied only to A32V caused a negligible response, while that stimulus only to A11 rapidly (in 0.86 s) induced a different beat pattern (not shown), repeating at 8.6 s. The response of A32V is dominated by the response of the inhibitory subpopulation oscillation, shown in Supplementary Figure S10. All subsequent results are in response to half-wave stimuli (Supplementary Figure S9B). A comparison of responses in A32V to stimuli to both A32V and A11 in the four standard frequency bands is shown in Figure 3. The stimulus frequency in each band is chosen to match the fundamental resonances listed in Table 1.

The A32V waveform transitions to a lower amplitude steady state after varying transition times, with a long delay of 12.5 s for a beta-modulated stimulus (Figure 3C) but only 1.6 s for gamma (Figure 3D). The gamma stimulus induces an additional long period, ramped pattern, repeating after 6.6 s (0.15 Hz), which is discussed below (Section 3.3). The theta stimulus, possibly modeling the multiple inputs of neighboring theta oscillators in the dominant spectral band, induces a long-lasting, low state that eventually (after 17 s) relaxes back to the resting state (cf. Supplementary Figure S11). The alpha stimulus appears to induce a disordered state (Figure 3B) of uncertain interest. Note that the theta stimulus was tuned to the resonance of A32V (5.3 Hz); the other theta resonance, for A46D (5.9 Hz), results in a fast (0.28 s) transition to a multi-beat waveform (not shown), possibly suggesting a different modulating role for A46D. Many tests indicated that consistent results were obtained when stimuli were applied simultaneously to both the in-hub, A32V, and to the in-connector, A11, suggesting a local circuit functioning as an AND logic gate. Network analysis and path tracing (Pailthorpe, 2024) also showed that both these nodes are the ultimate target of many sensory pathways. Stimulus to a single node was tested, e.g., application of the beta wave to A32V only induced a fast (1.8 s) transition to a smaller (by 8 mV) amplitude beat, while beta stimulus to A11 only produced a negligible response (~1 mV change). Generally, the average LFP for the cluster exhibited only small changes. The prominent response was for the local potential at A32V. As a final check, switching off the stimulus wave generally caused the oscillation to revert to its original state.

### 3.3 Response to reset stimuli

**Figure 4:**
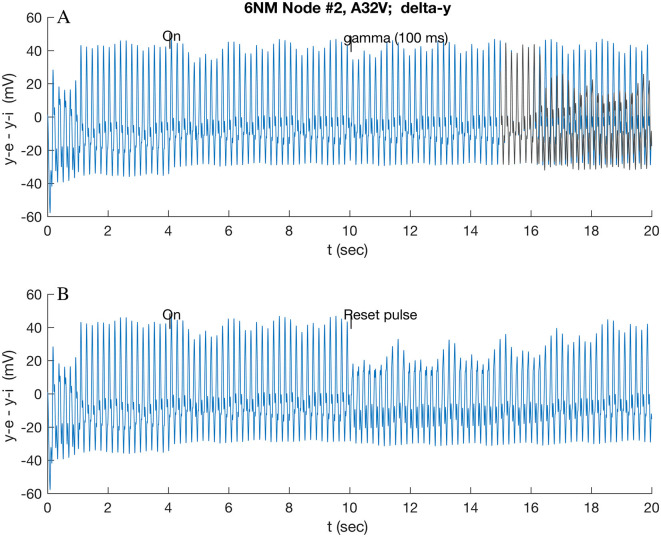
Response of A32V to beta band (16.0 Hz) modulated stimulus at 4 s applied to A32V and A11, followed by short reset stimuli applied at 10 s applied to all nodes: **(A)** 100 ms gamma burst; and **(B)** standard pulse function (cf. Section 2.5). In **(A)**, the original theta-induced transition at 16.5 s (cf. Figure 3C) is overlaid in yellow/gray for reference.

For the resting state cluster (cf. Supplementary Figures S7, S8), the dominant spectral power is in the theta band (63%) and then in the beta (21%) and alpha (14%) bands. Current pFC reviews (Lundqvist et al., 2018a,b) suggest that transients in beta and gamma bands have a role in working memory readout. Beta traveling waves have been observed to originate from pFC during task performance in macaques (Bhattacharya et al., 2022). To prove this, a beta wave was applied to A32V and A11 at 4 s, and then a second stimulus was applied 6 s later. The reset stimulus was applied to all nodes. Sensitivity was tested by also applying it selectively to single and pairs of nodes. Beta emissions from the cluster were not sought nor observed. The first beta stimulus (Figure 3C) appears to induce a latent switch that, after 12.5 s, transitions to a “low” amplitude state that can be interrupted within 0.1 s by switching off the beta wave at an intermediate time (e.g., 10 s), inducing a return to the original oscillation mode (not shown). That transition also is inhibited by a second, short stimulus, as shown in Figure 4. This transition is affected by a 100-ms gamma burst to the single nodes A32V, A10, or A46D but not by the stimulus of A11 alone.

A 100-ms gamma burst (Figure 4A) applied at 10 s (i.e., before the anticipated transition at 12.6 s) rapidly (~0.1 s) induces a reversion, within 1.0 s, to the initial state (overlaid in gray in Figure 4A). This short stimulus delivers little energy to the oscillators, so it needs to be close to in-phase with the excitatory potential *y*_*e*_ (of A32V) of the responding node to effect the transition; if it is out of phase, a proportionally reduced effect follows. By contrast, a simple pulse function stimulus, given by Equation (3) of Jansen et al. (1993) (reproduced in Section 3.5), interrupts the intermediate oscillation and initiates (within 0.1 s) the transition to the low state (Figure 4B), which then slowly relaxes to the original oscillation. These two stimuli have quite different energy content since a 100-ms gamma burst contains up to 6 positive peaks, each of which is approximately equivalent to a single pulse.

**Figure 5:**
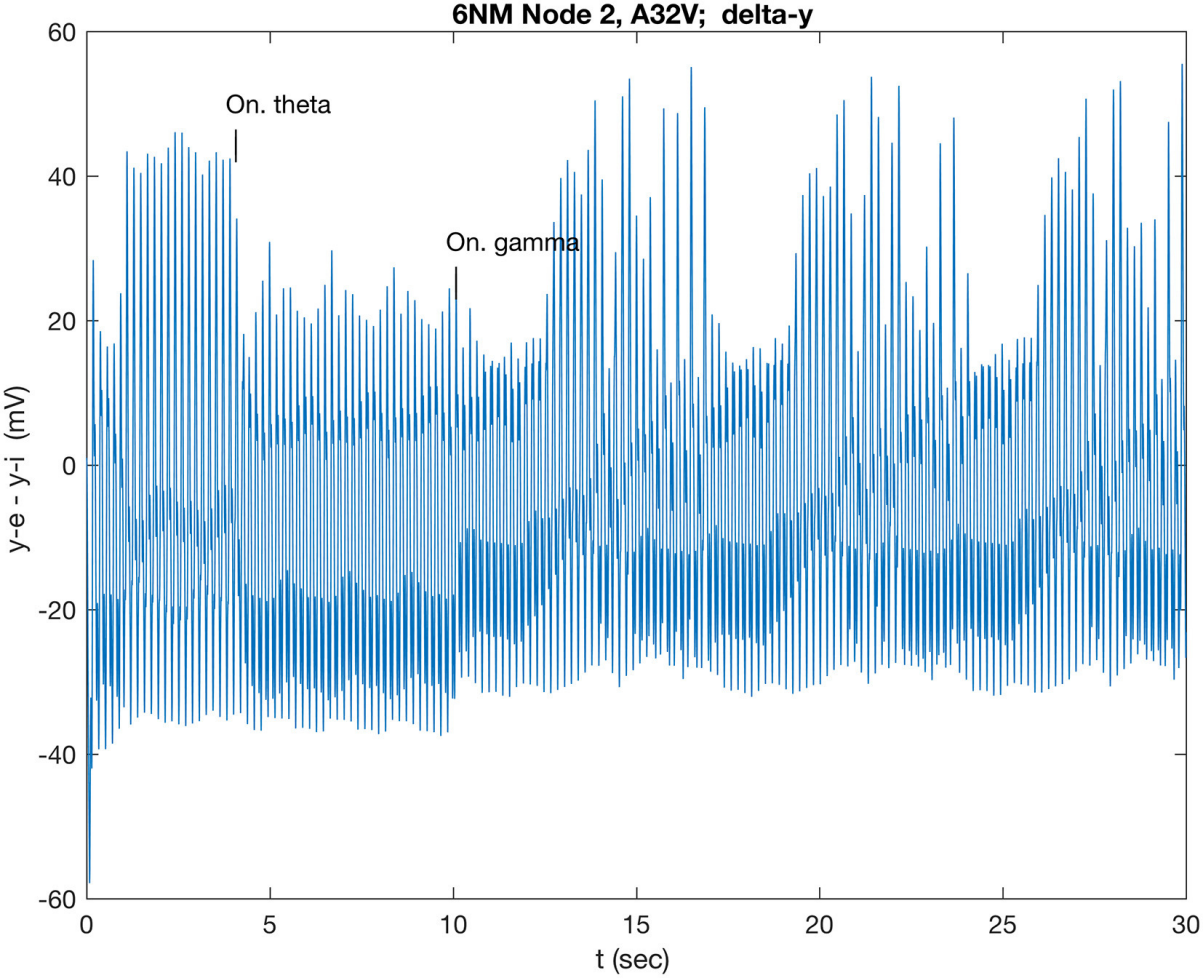
Response of A32V to theta band (5.3 Hz) modulated stimulus at 4 s, applied to both nodes 2 and 6 (A32V and A11) at 4 s, followed by a gamma wave applied at 10 s to all nodes.

Since the theta band dominates the power spectrum in the resting state, comparison simulations were run: a sustained and a short gamma burst were also applied after the theta-induced transition (Figure 3A). A continuous gamma wave applied at 10 s, after the theta wave induced low state had stabilized, induces a new oscillatory mode that repeated at ~6.7 s, as shown in Figure 5. By contrast, a 100-ms gamma burst had only a small effect on the theta-induced oscillation, even when care was taken to ensure that the stimulus was in phase with the *y*_*e*_ (A32V) oscillation.

Given the interest in gamma bursts, the results were investigated further. The system is a set of physical oscillators, and these transitions are likely to require sufficient energy input to drive them into a new state. Thus, longer gamma bursts and repeated short bursts that were phase-aligned to the positive parts of the theta oscillation were simulated. Recall that the theta-modulated dynamics spontaneously revert to the high amplitude state after 17 s (cf. Supplementary Figure S11). A single 500-ms gamma burst induced that reversion after 14 s, and a double amplitude 500 ms burst reduced that wait time to 12.3 s (figure not shown). Such a longer burst drives the oscillators with, and then against, the original theta-induced driving force. To avoid that, three 100-ms gamma bursts were constructed to phase align with only positive cycles of the theta oscillation that induced the transition after 13.9 s. This illustrates how gamma bursts, properly phase aligned, can deliver more efficient energy transfer and deliver a faster dynamical state transition of the cluster.

## 4 Discussion

Network analysis of the marmoset cortex structural linkage data identified a novel 6-node cluster in and around pFC. This is the dominant cluster in the marmoset cortex. In summary, this study implements a suggestion noted in the original study (Wilson and Cowan, 1972) to extend the classical WC/JR neural mass model to include a distribution of synaptic weights. The new voltage-to-rate transformation function is sharper than the usual sigmoid function. Available structural connectivity data for the marmoset cortex provides estimates of required parameters and guides the tuning of nodes to the standard frequency bands. Heterogeneity enters via linkage weights and the tuning of each node to a natural oscillation frequency. With synaptic weights and heterogeneity added, the NM model exhibits a variety of interesting dynamical responses and transitions. The results presented are for one set of parameters (Table 1). The simulations produced a balanced spectral density distribution (Supplementary Figure S8) dominated by the theta band and exhibited dynamical transitions in response to wave-like stimuli.

The resting state LFP of the cluster (Supplementary Figure S7A) is a composite of the component oscillations of the individual nodes (Supplementary Figure S7B) and exhibits a complex pattern of multiple beating oscillations. Wave-like stimuli were applied to the system to characterize its responses: the model produced sustained transitions in the dynamical state of A32V (Figure 3), with some new states lasting ~5–10 s, before relaxing. The new states comprised lower amplitude oscillations with variable beating patterns. Consistent with the observed role of beta waves in suppressing information (Lundqvist et al., 2018b), the simulated stimulus delayed the switch to a low amplitude state (Figure 3C). By contrast, gamma induced a new, repeating, ramped dynamical state (Figure 3D), possibly related to the observation: “gamma ramp-up coincides with working memory readout” (Lundqvist et al., 2018b).

To probe the system, further stimuli, based on experimental observations in the cortex, were applied to the model cluster. First is the observation of traveling waves in the visual cortex of an awake marmoset (Davis et al., 2019) during a task of visual detection of a moving target. The waves were in the low beta range (12 ± 4 Hz) traveling at slow speeds (≲1 m/s), representing unmyelinated axons. Their phase alignment was observed to be predictive of an excitable state during evoked responses. Second are observations of traveling waves in macaque pFC during a delayed match-to-sample working memory task (Bhattacharya et al., 2022). Alpha and beta power was observed to decrease during sample presentation, while beta and gamma power increased. Third, observations of beta and gamma bursts during working memory tasks in rhesus monkeys (Lundqvist et al., 2018b) suggested their control roles, as discussed above.

Together, these observations prompted simulations in which a wave stimulus was first applied, followed by a 100-ms gamma bust, which caused further dynamical transitions (Figures 4, 5). Theta and beta waves were chosen as the basic stimulus, with theta representative of a resting state environment due to inputs from neighboring areas and beta of the other possible control inputs. Beta wave stimulus followed by a subsequent gamma burst exerted control by interrupting the previously induced transition (Figure 4A). This is consistent with the suggested role of beta in suppressing information (Lundqvist et al., 2018b) and of gamma exerting further control on that process. The theta stimulus followed by a gamma burst induced a new dynamical state (Figure 5), illustrating how gamma bursts can control transitions between high- and low-amplitude oscillatory states. The importance of the simulations of the brief stimulus being phase-aligned with the base oscillation was noted above.

The current level of the NM model appears to only allow a small degree of heterogeneity, as illustrated by the parameter range in Table 1. Even so, the model can generate oscillations in the standard frequency bands and interesting transitions. Further refinements of the model are required to accommodate the range of the empirical synaptic weights. This could be incorporated via the gain constants, A and B (Equations 3–5), which reflect local synaptic weights in the neural feed-forward subpopulations, and via the feedback gain constants, C.

### 4.1 Other clusters in the marmoset cortex

A number of structural clusters in the marmoset cortex are described in my earlier study (Pailthorpe, 2024), with the pFC cluster being the most distinctive. It is compact and almost fully linked, with 29 of 30 possible internal links present; one-third of those have strong weights. Other clusters were also investigated amongst the remaining hubs and prominent connector nodes in the cortical network. Two candidates were an auditory group (AuA1, AuCPB, and AuRT) and a visual group (V3, V4, and V6). A motor-somatosensory group [cf. Supplementary Table S2 of Pailthorpe (2024)] was found to be more spatially dispersed and less tightly interlinked; another in-hub, TEO, was linked with the visual group. These clusters were weak, longer range, and less fully interlinked. Their linkage patterns were more tree-like rather than the tight, star-shaped patterns found in the pFC cluster. All these other clusters warrant further investigation.

### 4.2 Sensory inputs to and motor links with the cluster

All six nodes in the cluster received inputs from the visual cortex, mostly from V2 via TPO. Other links, via MST or Opt or direct from V2 or V4, are weaker. The strongest weight pathways from the auditory cortex are from AuA1 and AuCPB via TPO to A32V and A11, consistent with the observations of Dorsal and Ventral auditory pathways (Schreiner and Winer, 2007) and the sensitivity of A32V and A11 to stimuli in the simulations. The somatosensory areas (cf. Section 4.1, above) have direct links to A10 and A11. There are reciprocal links between A10 (the out hub) and the pre-motor area A6DR, which has a strong input to A46D in the pFC cluster. The strong links to A46D, along with its simulated responses, suggest it may have a role in modulating pFC cluster responses.

### 4.3 Bursts, avalanches, coherence, and transitions

The present study demonstrates dynamical transitions in localized clusters of cortical areas modeled as simple oscillators. On a larger scale, two studies shed light on bursts, transitions, and synchronization and are accompanied by significant literature. The first was an experimental investigation of spontaneous LFP oscillations in rat somatosensory cortex measured by micro-electrode arrays (Giresch and Plenz, 2008), which observed beta and gamma bursts nested within theta oscillations. The oscillations were coherent between nearby areas, with the synchronization following a power law across distances up to 1.6 mm, and the nested oscillations being organized as avalanches. In the present study, higher frequency, brief stimuli needed to be phase-aligned with the basic oscillation if they were to effect a dynamical transition. Thus, 4–5 gamma peaks could fit within a single theta positive oscillation, enhancing the underlying oscillation and producing the complex waveform consistent with the experimentally observed beta-gamma/theta nesting.

The second study was a theoretical and computational study of a modified Wilson-Cowan model applied to a 2D lattice of diffusively interacting nodes driven by noise (Di Santo et al., 2008). The lattice was rewired to allow a small fraction of long-range connections. It included a model of synaptic plasticity and some heterogeneity via modified node activity levels. A critical point in the cortical dynamics, indicative of a phase transition in the synchronization, was observed by tuning the baseline level of a synaptic resource parameter. Such a global transition is in contrast to the more localized up-down state transitions found herein. That study also predicts avalanches, as observed experimentally. The synchronization transition was observed in an extended system of size 64^2^-512^2^ oscillators. Another relevant study is of a system of 80 identical NMs, each connected to 20 neighbors connected in a Watts–Strogatz network, with a specified interaction strength (Kazemi and Jamali, 2022). The system exhibits a synchronization transition as coupling strength is increased, and the transition point shifts with noise stimulus strength.

In each of those studies, the system has a large number of interacting entities, and the transition is driven by interaction strength, while the present study was of a local cluster of six oscillators coupled by the fixed experimentally specified interaction weights and driven by wave-like stimuli. The small size is far removed from the thermodynamic limit of large systems where phase transitions and critical phenomena are usually observed. The transitions observed herein are between up-down states of high and lower amplitude. It would be worth searching for suitable measures of synchronization and their transitions in small groups of NM oscillators and in the pFC cluster. Small groups of oscillators are known to synchronize, thus encouraging such a search. For example, the synchronization of two pendulum clocks has been known for centuries [reported by Huygens in February 1665 (Strogatz, 2003, p. 106)], suggesting that synchronization may be possible in small clusters of oscillators. Whether transitions occur is less certain. The roles of localized up-down states, larger scale synchronization transitions, and the interplay between system size, variability of interactions, and stimuli, all warrant further investigation. The global model of di Santo et al., drawing on the statistical physics of critical phenomena, is more sophisticated than the localized clusters of simple oscillators and so provides a larger perspective on the present study.

### 4.4 Strengths and limitations

The classic WC/JR model has been extended to incorporate the distribution of synaptic weights, leading to a sharper voltage-rate transformation function (Equation 6; Supplementary Equation S5). Heterogeneity has been included via experimentally available linkage weights. Together, these induce interesting dynamical behavior.

The heart of the WC/JR model is a simple, critically damped oscillator wholly driven by external inputs, so it is amenable to intuitive physics to guide its further development and interpretation. This makes clear that brief stimuli, such as gamma bursts, need to be phase-aligned with the underlying oscillations for the stimulus to deliver sufficient energy to drive the oscillations to a new dynamical state. Such phase alignment is reminiscent of the observed nesting of beta/gamma-theta oscillations (Giresch and Plenz, 2008).

A key limitation of NM models is that a number of scales are unclear (cf. Supplementary material 2.7): the spatial extent of a neural mass, the relationship between linkage weights and the synaptic weights, and the relationship of voltage output to measured LFP. The model developed herein can benefit from the inclusion of a model of synaptic plasticity (Tsodyks and Markham, 1997; Di Santo et al., 2008), e.g., via a generalization of the feed-forward and feedback constants A, B, and C (cf. Equations 1–5, Supplementary material 2.2). A biologically plausible way of incorporating synaptic plasticity in the WC/JR model, that avoids arbitrary parameterization, may yield informative dynamical responses. It is unclear if small clusters of NMs can produce synchronization transitions (cf. Section 4.3); more work would be required to establish the cluster size that can produce such.

### 4.5 Future work

This study highlights several areas in and near pFC that have interesting roles in local dynamics. Area A32V, the major network in-hub in the marmoset cortex, has a distinctive response to stimuli that warrants further investigation. A11, a connector node and marginal in-hub, is a target of numerous sensory inputs. A46D was sensitive to reset stimuli, indicating a possible role in modulating pFC function; it receives medium-strength inputs from the auditory cortex. The availability of high-density electrode arrays that may be able to probe LFP at the single area level offers an opportunity to study these areas in detail. The new structural data for marmosets at a finer spatial resolution (Watakabe et al., 2023) provides an opportunity to extend the present study.

## 5 Conclusion

The model constructed herein emerged from a network analysis of the experimentally observed marmoset connectivity data, in contrast to being assembled to address specific observations. The simulations map out some basic dynamical transitions in a cluster of areas around marmoset pFC in response to wave-like stimuli. The dominant transitions were found in area A32V, the major network in-hub in the marmoset cortex. While all nodes showed some response, the prominent transitions occurred when A32V and A11 were both stimulated. The new dynamical state could then be modified by gamma stimulus of any one of A10, A11, or A46D.

The range of response illustrates the possibilities that emerge from heterogeneous models of NM tuned to available connectivity data. This study demonstrates that multiple, simple processes can generate dynamical transitions in time windows consistent with those of working memory. It provides a framework in which specific events can be placed to generate biologically relevant processes. It also illustrates how local dynamics might relate to known large-scale nested oscillations and coherence. Computational studies can thus expose the inner workings of the oscillatory dynamics of neural assemblies to build insight and suggest experimental investigations.

## Data availability statement

The original contributions presented in the study are included in the article/Supplementary material, further inquiries can be directed to the corresponding author. The Matlab codes presented and used in this study are attached, or can be found at https://github.com/BrainDynamicsUSYD/MarmosetCortex/.

## Author contributions

BP: Writing—review & editing, Writing—original draft, Visualization, Validation, Software, Resources, Project administration, Methodology, Investigation, Formal analysis, Conceptualization.

## Footnotes

^1^MATLAB, Mathworks Inc. Waltham, Mass.
