# Supplementary material for: Simulated dynamical transitions in a heterogeneous marmoset pFC cluster
Source: Front Comput Neurosci. 2024 May 28;18:1398898. doi: 10.3389/fncom.2024.1398898 (PMC11165126; doi:10.3389/fncom.2024.1398898)
Supplement: Supplementary file 3 [file Data_Sheet_3.PDF]

Supplementary Material

Simulated dynamical transitions of a heterogeneous Marmoset pFC cluster

Bernard A Pailthorpe

Correspondence: Bernard.Pailthorpe@sydney.edu.au

1 Supplementary Data

The 6x6 adjacency matrix of link weights, measured as LNe [Pailthorpe 2024], is attached as file Adj6x6.csv.

The 6x6 matrix of link distances (mm) is attached as file Dist6x6.csv.

2 Supplementary Text

2.1 Voltage to Rate Transformation function.

A core part of the original WC and JR analysis of neural masses was the specification of the voltage to rate transformation relationship. This appears on the right side of eq. 3-5 and affects the external inputs to the NM. It is the key source of non- linearities in the model. The original analysis [Wilson and Cowan, 1972] canvassed two options in converting the buildup of presynaptic potential to generate a neural response (eg. spike): 1) a distribution of individual neural thresholds, $D(v_0)$, in a neural population with a constant number and weight of synaptic inputs; or 2) a distribution of the number or weight of input synapses per neuron, C(w), in a neural population with the same threshold, v_0. In each case the distribution is taken to have the standard normal or Gaussian form. The usual focus is on the first option only – with the formal analysis yielding an error function (erf) dependence on voltage. That is well approximated by the familiar and convenient sigmoidal functional form that has been used in almost all subsequent studies. However measured firing thresholds vary by only 10's % [Henze and Buzaki 2001, Yu et al 2008], while connectivity data, of number of links and link weights, can vary by 2-3 orders of magnitude, as with the present data.

For option 1 the neural population is subject to an applied voltage v(t); the fraction of excited neurons, i.e. those above each neuron's threshold voltage v_0 at constant synaptic weight w, is

$$f(v) = \text{fraction}(\, v > v_0 \mid w)$$

$$= \int_0^{v(t)} D(v_0)\, dv_0$$

$$= \frac{1}{\sqrt{2\pi}} \int_0^{v(t)} e^{-(v_0 - v_{av})^2 / 2\sigma^2}\, dv_0$$

where exp is the exponential function, v_{av} is the average firing threshold and σ is its standard deviation. This form implicitly assumes that the constant synaptic weight w is 1. With a simple change of variable this yields

$$f(v) \quad = \quad \frac{1}{2}\left[\operatorname{erf}\left(\frac{(v(t)-v_0)}{\sqrt{2}\,\sigma}\right) - \operatorname{erf}\left(\frac{v_0}{\sqrt{2}\,\sigma}\right)\right] \qquad \text{(S1)}$$

where erf is the error function which is readily available [Arfken 1970] and implemented in computational environments such as Matlab or Python. The voltage to rate transformation function appearing in eq. 3-5 is formed by multiplying by a maximum rate q_m, usually taken as 5 Hz [WC, JR]. The erf function has a sigmoidal shape, resulting in the convenient approximation:

$$S[v(t)] \quad = \quad \frac{2\,q_m}{\left[1+ e^{(-(r(v(t)\,-\,v_0))}\right]} \qquad \text{(S2)}$$

which is the usual form in the literature, with slope now written as $r = 1/(\sqrt{2}\,\sigma)$. A simple extension of the above analysis generalises it to non-unit average synaptic weight, resulting in:

$$S[v(t)] \quad = \quad \frac{2\,q_m}{\left[1+ e^{(-(r(w_{av}v(t)\,-\,v_0))}\right]} \qquad \text{(S3)}$$

Where w_{av} now is the average synaptic weight in the neural population.

For option 2 the neural population has constant threshold v_0 and a distribution of synaptic weights, again subject to an applied voltage v(t). Now the neural population has a distribution of the number or weight of input synapses per neuron, C(w). Repeating the above analysis, the fraction of firing neurons is an integral over the distribution of synaptic weights at constant firing threshold voltage v_0:

$$f(v) \quad = \quad \text{fraction}(\,wv \;>\; v_0 \,|\, v_0\,)$$

$$= \quad \int_{v0/v(t)}^{\infty} C(w)\,dw$$

Note that the unusual voltage dependence arises from writing the firing criterion (sufficient excitation) as $w > v_0/v(t)$ and integrating over the synaptic weights. Thus

$$f(v) \quad = \quad \frac{1}{\sqrt{2\,\pi}} \int_{v0/v(t)}^{\infty} e^{-(w-\,w_{av})^2/2\sigma^2}\,dw$$

where v_0 is the now constant threshold voltage; and w_{av} is the average synaptic weight in the neural population with σ as its standard deviation. The result now is:

$$f(v) \quad = \quad \frac{1}{2}\operatorname{erfc}\left(\frac{(\,{}^{v_0}/_{v(t)} - w_{av})}{\sqrt{2}\,\sigma}\right) \qquad \text{(S4)}$$

where erfc is the complimentary error function, (1- erf), also readily available [Arfken 1971] and in Matlab; and again $r = 1/\sqrt{2}\,\sigma$. Note that erfc(v) is continuous at v = 0 but has a discontinuous slope

there, and with the appearance of 1/v(t), care needs to be taken to ensure that v(t) is strictly positive, as is the rate function. The resulting voltage to rate transformation function is:

$$S[v(t)] = q_m \,\mathrm{erfc}\left(-r\left({}^{v_0}/_{v(t)} - w_{av}\right)\right) \qquad \text{(S5)}$$

Eq. S5 is the form adopted in this study. It is worth noting that synaptic weight distributions may also have a log-normal distribution [Pailthorpe 2014, Robinson et. al. 2021, Buzsáki and Mizuseki 2014], and that the above analysis can be so extended. The figures below compare the three forms.

< Figure S1 >
< Figure S2 >

2.2 Parameter choices.

The coefficients A and B (eq. 2-5) quantify the feed forward synaptic strengths, while C_1 - C_4 describe the feedback links to the excitatory and inhibitory populations; and originally described as the "average number of synapses per cell" [Jansen and Rit 1972]. Physiological constraints indicated the ratios $C_2 = 0.8\, C_1$, $C_3 = C_4 = 0.25\, C_1$ [Jansen and Rit 1995], as adopted in most studies and herein. Setting $C = C_1$ eliminates three parameters. The free parameters of the WC/JR neural mass model are A, B, C, τ_e and τ_i; along with q_m, v_0, r, $w_{av\text{-}in}$ and $w_{av\text{-}out}$ associated with the sigmodal v–rate transformation. The first 7-8 parameters are standard, while the two w_{av} are introduced here. The present analysis generally follows the standard choices already established in the NM literature [Wilson and Cowan 1972, Jansen and Ritt 1995]. Earlier studies [Wendling et. al. 2000, David and Friston 2003] have varied C, τ and r to tune the characteristic frequency of a NM. The standard values of q_m = 5 Hz and v_0 = 6 mV are used here. Typically lower C (~135-200) and r (0.4) along with higher τ (20 ms) produced lower frequency oscillations (theta, alpha bands), while higher C (>300) and r (0.5 , 0.56) along with lower τ (10 ms) produced higher frequency oscillations in the beta or gamma bands. As noted, the form of eq. 1, 3-5 aids in tracking the balance of driving forces. A/τ_e and B/τ_i set the relative scales of stimuli for the excitatory and inhibitory sub-populations (eq. 3,4). Some authors have noted the need to retune A [Wendling et. al. 2000] in order to achieve oscillation. Here A and B are left at their original values: A = 3.25 mV, B= 22 mV [Wilson and Cowan 1972, Jansen and Ritt 1995] and only C, r, τ_e and τ_i are tuned. For the higher bands the usual choice ($\tau_i > \tau_e$) does not lead to oscillations, since the inhibitory drive is too low. Typical values that lead to oscillation were found with $\tau_e \cong \tau_i$ - without resort to retuning A. If $\tau_i > \tau_e$ was to be preserved the equivalent tuning would be to decrease A or increase B. Near equal τ's also is consistent with the original observation that τ arises from time constants of the membrane resistance/capacitance and distributed delays averaged over the dendritic networks [Jansen and Rit 1995]. It is credible that these might be similar in the excitatory and inhibitory neural sub-populations. This is distinct from the neuron level time constants, associated with ion channel opening and closing, used in multi-neuron simulations (cf. 2.6 LIF, below).

2.3 Sensitivity to parameters

Most parameter (A, B, C, q_m, v_0, r) values were chosen following previous studies discussed above and not varied. The present simulations showed that the next most important inputs were the node size and inter-area link weights. Sensitivity to these inputs was tested by systematically adjusting link weights singly and in small groups. The strongest links (weights ~ 5-10 k) are robust to 10-20% variations, as are the weakest links (weights $\leq$ ~10-20) which can be eliminated without significant impact of observed responses. In general, modest variations of arbitrary links in the 6 node network has little effect on system dynamics. If groups of three links (10% of the 29 present) are adjusted randomly by ± 25-50% then the individual dy's and net LPF adopt a simple sin form; or occasionally oscillations in nodes 2, 5 and 6 may be extinguished. The overall balance of link weights, as measured for the marmoset cortex, appears to be required to generate the complex, beating voltage waveforms found.

To explore the parameter landscape two set of test simulations were performed: the voltage-rate response function slope, r was varied, while retaining the derived distribution of mean weights (cf. Table 1, S1). Then w_{av} was varied, keeping r as the derived vector. First, the slope, r, was set to its mean value for all nodes, and also to mean +- one standard deviation, all resulting in only small effects in the oscillation amplitudes (~ 2-4mV) and waveforms. The mean synaptic weights of w_{av}, are a more sensitive parameter: setting all to their mean value extinguished all oscillations. The largest nodes (2 & 6) are robust to 25% reductions, resulting in <10mV variations in voltage amplitudes. While individual node waveforms are similar, their phase relationships do change, resulting in variable overall waveforms.

Choosing all w_{av} = 1 (close to the usual sigmoid curve) resulted in simple sin oscillations at 6.0 Hz for all nodes. Constant w_{av} (eg. at the mean of all values) across nodes extinguished oscillations, while variations of a single value (for nodes 2, 5 or 6) by -25% changed the wave form but still allowed sustained oscillations; and all w_{av} = 6 extinguishes all oscillations. Those values represent the viable range of w_{av} values that can sustain oscillations. These simulations highlight that oscillations of nodes 2 (A32V), 5 (A46D) and 6 (A11) are sensitive to perturbations in parameters. The observed distribution of link weights between areas and mean synaptic weights within areas together induce the complex waveforms observed, that arise from constructive and destructive interference of the contributing waveforms [Halliday et. al. 1997]. It is these that have the potential to carry information [Einevoll 2013].

2.4 Link weights

The adjacency matrix, of weighted link strengths, for the six node cluster is attached as file Adj6x6.csv.The Marmoset brain Atlas [Paxinos et. al. 2012] and associated volume images enable calculation of the volume of each anatomical area by counting labelled voxels. The calculated centre of mass, or centroid, of each area's voxels was taken to be the node coordinates. Neuron density and cortical thickness [Atapor et. al. 2019] and enable calculation of the number of neurons in each area (cf. Table 2). The reported structural connectivity, or adjacency, matrix (http://marmosetbrain.org) enable calculation of the link weights [Pailthorpe 2024] used here, reported as LNe, so w =1 corresponds to a single labelled neuron. The full adjacency matrix for the 116 areas of the cortex is available [Majka et. al. 2020]; for the six nodes of the cluster it is attached at Supplementary Data.

The average weight per local link (in, out), w/k, in the cluster was used as a guide to estimate the parameter w_{av} in the synaptic function S[v(t)] – cf. eq. 6. The familiar exponential decay of link weights with distance [Pailthorpe 2024] was observed for the Marmoset data, so only local link data, within the six node cluster, were used. Summary data are presented in Table S1: number of links, k and sub total of link weights w (in & out) are for local links only – ie. within the cluster. There are several choices for the ratio w/k: in, out, average, maximum of in and out link data; and it is unclear which would be appropriate to use; all were tested in this study. The ratio reported in Table S1 is the average weight per link (both in & out): $(w_{in}/k_{in} + w_{out}/k_{out})/2$; this is rescaled ≥ 1 (cf. Fig S2) and used as w_{av}. Note that for A32V the reported ratio is likely an underestimate: The single link A10 – A32V has weight 9.1×10^3, 57% of the total of local in weights (16.1×10^3) to A32V, suggesting that the averaging skews the ratio lower; similarly for A46D. Thus higher w_{av} was explored for nodes A32V and A46D in the simulations.

< Table S1 >

Another option is to consider the weight of links per neuron as a measure of synaptic weight, as presented in Supplementary Table S2.

< Table S2 >

Data on external links of the cluster to and from the rest of the cortex are presented in Supplementary Table S3. These exclude the links reported in Table S1. They give an indication of the significance of each node within the context of the whole cortex.

< Table S3 >

Another relevant measure available from the data is the link weight per neuron (Table S2). Comparison of the optimum w_{av} used in the simulations (Table 1, for trial #6) with estimates of w_{av} derived from the marmoset data (Table S1) is presented in Figures S3 and S4. Linear trends of varying quality are evident. Fits (Fig. S3) of trial w_{av} vs weight per in link data yield $R^2 = 0.78$, average of w/k(in, out) ($R^2 = 0.52$); and of trial w_{av} vs in weight per neuron (Fig. S4; $R^2 = 0.70$) and trial w_{av} vs out weight per neuron (not shown; $R^2 = 0.19$). A9 and A32V are consistent outliers (and possibly A11) using w/k. While A32V (the main in hub in the cortex) is more consistent with a linear trend using w-in/neuron. The large scatter evident with w-out/neuron suggests it is less relevant. The values of listed in Table 1 were used herein. They are most consistent with the experimental in weight per neuron.

< Figure S3 >

< Figure S4 >

2.5 Frequency band assignment

Here frequency band assignment to the 6 nodes emerged from exploratory simulations of 1, 2, 4 and 6 node clusters, along with the LIF simulations of NM models of varying size (number of neurons, N). Studies (not reported) of two oscillators (cf. eq. 1) linked by linear coupling indicated the range of frequency shits induced by link weights. For two oscillators tuned to theta and alpha frequencies respectively, increasing link weights (0.1 – 1.0 (arbitrary units)) induces small frequency decreases (~2-4 Hz) in both nodes. Stronger (> 2.0) links eventually extinguishes oscillations due to the critical damping. Earlier studies [David and Friston 2003] explored the range of C, τ_e, and τ_i, for

characteristic frequency bands. Here additional data is available from the link weight statistics: $w_{av\text{-}in}$, $w_{av\text{-}out}$ and r. Exploratory simulations of one or two NM (isolated, or unit links) sought the dominant node frequency f of peaks in the power spectral density derived via fast Fourier Transform (fft) of the output LPF ($dy = y_e - y_i$). Characteristic peaks in each band, when present, were fairly consistent across parameter searches. In summary: in the theta (4-8 Hz) and alpha (8-12 Hz) bands the feedback strength C is small (C ~ 180 - 200), the time constants τ_e and τ_i are long (~ 10 – 25 ms), with the resulting dominant frequency decreasing as w_{av} increased (w_{av} ~ 1-3), and increasing as r increased (r ~ 0.45-0.5). Secondary, much weaker, peaks were also present. Searching in the beta (12-30 Hz) and gamma (30-100 Hz) bands required C larger (C ~ 250 - 350), shorter τ_e and τ_i (~ 5 - 10 -15 ms), and larger w_{av} (~ 3 - 5). Many parameter choices resulted in no oscillations, so trends were harder to discern; and gamma oscillations only emerge in a restricted range of parameter values, found after some searching.

Typical parameter values, for a single NM, viable for each frequency band, are listed in Table S3. Other values, listed in Table S4, illustrates sensitivity to parameters. Many combinations that resulted in no oscillation are omitted.

< Table S4 >

< Table S5 >

Introducing extra links between nodes in these small clusters resulted in small (< 1.5 Hz) frequency shifts of spectral peaks. It emerged that r was less important, so r = 0.5 was adopted throughout. The 4-node sub-cluster, comprising A10, A9, A46D and A11, is generally more compact and is a natural precursor to the 6-node cluster. This provided a test case for tuning the four standard frequency bands of anatomical areas. Test simulations of the 4 NM in linear, square and star shaped linkage patterns, with α−α, α−β, α−γ, β−β, etc. neighbours, showed which patterns yield stable oscillations and spectra. Frequencies found for these 4 nodes yielded a preliminary band assignment, listed in Table S6.

< Table S6 >

For comparable parameters some nodes in Table S5 show slightly different frequency compared to Table 1 due to the varying link patterns and weights. Overall 10 trial combinations of parameters for the six node cluster, involving. small variations, produced quite different LFP dynamics. Two trails (#2, 6; cf. Table 1) of those ten produced the transitions reported herein; the others did not display transitions in response to the wave like stimuli. Results reported are for trial 6, with parameters listed in Table 1.

2.6 LIF simulations of a Neural Mass

Output of neural masses have also been studied by simulating populations of excitatory (80%) and inhibitory (20%) neurons, e.g. by using a simple Leaky Integrate and Fire (LIF) model of a neuron [Mazonni et. al. 2008, 2015]. Neurons were connected randomly so that a fraction (p =02, 0.4, 0.6) of unit weight links were formed. The available c codes are linked into Matlab via its mex interface. Here the conductance based version (COBN) of the model and codes was used to study the size dependence of the resulting LFP dynamics. The fraction of gamma (40-100 Hz) power in the total

spectrum was used as a simple measure of high frequency oscillations (Figure S5), rather than plot spectral peaks which would yield a more cluttered graph. LIF simulations indicated that the high frequency fraction of the power spectrum increased with N up to N ~ 400, and was constant or decreased slight with increasing N beyond that, out to N =10,000. The effect was more pronounced for a small fraction of links (cf. 26% for marmoset data), along with low noise. This result is explained as follows.

< Figure S5 >

Basic statistics [McQuarrie 1973] indicates that fluctuations in large systems of size N scale as 1/√N. That is consistent with the decrease for large N in Fig. S5. For small systems that result does not apply. In the present case the balance of excitatory and inhibitory stimuli to relatively few linked neighbours determine the frequency of spiking and ultimately the frequencies present in the total LFP output. For the LIF system N = Nexcit + Ninhib, with an 80-20% split. Connection types are: excit – excit; excit – inhib; inhib – excit; and inhib – inhib, each with probability: 0.2, 0.4, 0.6. Each neuron is exposed to a level of random noise (2, 4, 6 Hz). Connection probabilities are: 0.2, 0.4, and 0.6. The central variables in the simulation are Firing Rates: FR-e and FR-i. Essentially for the LIF system to exhibit high frequencies (ie faster FR) the excitatory population needs more stimuli, either from other excitatory neurons or the external noise. Yet for low N, Ne is also lower and, with lower connection probability, fewer excit – excit links exist, and FRe is smaller, but still present, leading to LFP in the theta and alpha bands. For small increases in N there are more excit – excit, but not too many inhib – excit, links, thus increasing FRe and leading to more spectral power in the gamma band. More links and/or noise increase FR and leads to more spectral power in the gamma band. As N increases further there are more inhib – excit links, suppressing FRe; and more inhib – inhib links increasing FRi, thus slowly reducing high frequency components of the output LFP. The linear increase in Fig. S5, for small N, compares with a similar trend of computed node frequencies vs N (cf. Table 1). While suggestive that is not conclusive since N for anatomical areas is very large (~ 10^5), while for mini columns it is of order 100's as in the linear regime in Fig S5. This begs the question of what scale NM models are applicable to?

2.7 Scale of model.

The first scale related question concerns the size of the cluster. For the selected six nodes the in and out links are shown in Fig. 1, computed for link distances < 3.5mm. The next two closest nodes (A8aD and A8b) add 5 out links and 5 in links to credibly form an 8 node cluster. Numerous simulations of that cluster (not reported) exhibit generally similar behaviour to that reported here. The 29 links internal to the 6 node cluster have a total weight of 42,578. The 5 out links to A8aD and A8b have total weight 4,090, while the 5 in links total 8,326. Fig 1B shows 13 additional in links from nearby nodes, with total weight 11,218. It's a matter of judgement where to draw the boundary. The 6 nodes have two thirds of the total link weight internal to that cluster, with one third to nearby nodes. The general assumption in neural mass modelling is that inputs from such local nodes are captured in the noise input. The present study provides some quantification of that assumption.

Other questions of appropriate scale in NM models enters in two places: the spatial extent of a neural mass, the unit studies by the mesoscopic models used herein; and the scale of synaptic weights. The original WC and JR models were developed for cortical columns, a fundamental unit of cortical structure [Mountcastle 1955, 1997], with diameter (i.e. scale) of order 0.5 mm and containing about 10^4 neurons in rodents (in SSs, barrel field) [Markham et. al. 2004, Keller and Carlson 1999, Lubke and Feldmeyer 2007]. Although widely discussed there is a degree of ambiguity and variability in

defining cortical columns based on their structure or function, and the generality of the concept beyond the sensory areas was the cortex was questioned [Molnar 2013]. Recent data from marmoset confirm the presence of larger columnar patches with an orderly spatial distribution and diameters of 0.67 - 0.84 mm in dlpFC [Watakabe 2023]. Repeated patterns within columns has led to the further concept of mini columns [Mountcastle 1997] comprising ~20-30 mini columns of diameter ~ 0.05 mm [Innocenti 2010, Table 2]. Anatomical areas, the network nodes considered herein, are larger, contain many cortical columns and receive more afferents. The six node cluster comprises anatomical areas with volumes in the range 2.7 (A32V) to 24 (A10) mm^3, corresponding to linear scales of 1.8 - 3.9 mm – being the diameter of an equivalent cylinder spanning the cortical sheet. Such a volume might contain 30 - 190 cortical columns (cf. Table S7), which in turn may contain mini columns.

< Table S7 >

2.8 Resting state of models

For comparison with the results in the main text, the output of the classic sigmoid model (cf. eq. S2) is presented in Figure S6, where the identical nodes are tuned to the beta band (cf. Table 1), with r = 0.5; and no w_{av} effect; i.e. homogeneous nodes. Links between nodes have varying weights given by the adjacency matrix.

< Figure S6 >

Fourier analysis of the LFP waveform, after transients, confirms a sinusoidal waveform of frequency f = 16.1 Hz, in the beta band, with a 10.7 s beat (0.094 Hz). The power spectral density is 99.7% in the beta band and 0.3% in the gamma; the latter peaks are at the first and second resonances of the fundamental frequency. The overall waveform is the results from constructive and destructive interference of the component oscillations. Plots of the individual output dy(t) for each node, shown in Fig. S6b, exhibit the same frequency with varying amplitude and phase. That is driven solely by the link weights and signal delays. The more complex model (eq. S1) yields the richer waveforms presented in the main text due to summing more variable individual oscillatory waveforms.

For the full model (cf. Main text; parameters in Table 1; eq. S5) the LFP output and component waveforms are shown in Supplementary Figure S7.

< Figure S7 >

With the more complex transformation S[V] and heterogeneous nodes the individual oscillation are slightly more complex, of differing frequencies, and combine to form a richer LFP waveform. Its component frequencies are listed in Table 1. The power spectral density comprises: theta band 63%, alpha 14%, beta 21% and gamma 1.7%. That spectrum calculated by Fourier transform is plotted in Supplementary Figure S8. The small system size produces only a few discrete peaks, in contrast to the continuous spectra observed in experiments for large scale model systems.

< Figure S8 >

While there are numerous reports of EEG in marmoset, the author did not find detailed spectra with which to compare the present results. Cortex-wide observations of EEG and LFG indicate a preponderance of power in beta, alpha and theta bands, somewhat consistent with the present simulations localized at pFC. Examples include: Whole scalp recording of EEG after vapour exposure showed unquantified peaks in theta and beta (~ 13 Hz) bands, cf. Fig 1 of [van Helden et.

al. 2004]. LPF recording at the left occipital cortex of marmoset in response to visual stimuli showed a spectrum with a beta peak at 13.4 Hz (cf. Fig 3F of [Schwenck et. al. 2022}), consistent with the beta peak in Fig. S8. Separately, [Konoike et. al. 2022] applied gamma stimuli to several species, including marmoset, with time -frequency analysis of evoked potentials showing enhanced spectral peaks at the stimulus frequencies.

2.9 Stimulus and Response

Different sized clusters clearly have varying outside surface areas of interface to the remainder of the cortex. Thus the quantity of noise input to the neural masses modelling the cluster may vary. To check if this may affect the results simulations were conducted on the 6-node pFC model (parameters tuned to yield alpha band LFP output) with varying noise inputs with scale 0.1, 1.0 and 10.0, being the standard deviation (sd) of Gaussian noise. In the each case the range of noise varied by about 3 sd and could equally likely be negative. For the two orders of magnitude range of noise inputs the cluster output (LFP) varied at most by only 1 mV across that range. In all simulations Gaussian noise of zero mean and unit variance was applied to all nodes.

A stimulus pulse train was constructed comprising a 100 Hz constant pulse rate modulated by a sin wave in one of the theta to gamma bands. The stimulus, being a pulse rate, needs to be strictly positive; that can be ensured by included a sufficient offset bias, or by a rectification process that may be full wave (converting negative amplitudes to positive), or half wave (eliminating the negative phases), as illustrated in Figure S9. In the full wave case the frequency is effectively doubled, and typically stays in the same band. In the half wave case the amplitude needs to be doubled (to 200 Hz) to ensure a constant energy input into the cluster oscillators and thus facilitate fair comparisons. For the biased case the amplitude needs to be adjusted (typically ~25%) to ensure constant energy input. Generally, two options (biased, half wave) produce similar responses, while the full wave stimulus, at double frequency, is distinct (cf. Fig. 2, S8a). To avoid going into the question of possible mechanisms, involving multiple synapses and dendritic networks, it is simplest to use the half wave form since this preserves the intended frequency and a positive stimulus. That was used in all reported simulations; and stimuli were applied to all nodes or to selected nodes, pairs, etc. as indicated in the text. All stimuli were turned on at 4.0 s, well after transients have settled down and applied either for the full simulation run (20 s) or turned off after 6 or 10 s.

Typically node #2 (A32V, the major in-hub) shows the most pronounced response (cf. Results), due to suppression of oscillation in the inhibitory population, as illustrated in Figure S10. For the other nodes the output dy(t) steps up slightly (< 2 mV).

< Figure S9 >

< Figure S10 >

The primary wave induced transitions (Fig. 3) were investigated beyond the 20 s simulation run time, as a check. Only the theta wave modulus stimulus exhibited a change at longer times. The theta (half wave – cf. Fig. 2a) induced rapid transition to a low state (Fig. 3a) was long lasting but not permanent as illustrated in Figure S11, which shows the transition of node A32V at 4 s to a low amplitude oscillation. then relaxes back to a higher amplitude oscillation after 17s. Note that after a further 15 s the new state exhibits a slight beating of amplitude.

< Figure S11 >

2.10 Supplementary References.

Buzsáki G, Mizuseki K. The log-dynamic brain: How skewed distributions affect network operations. Nature Rev. Neurosci. 15:2 64. 2014

Einevoll GT, Kayser C, Logothetis NK, Panzeri S. Modelling and analysis of local field potentials for studying the function of cortical circuits. Nature Rev. Neurosci. 14: 771-785. 2013. doi:10.1038/nrn3599.

Keller A, Carlson GC. Neonatal whisker clipping alters intracortical, but not thalamocortical projections, in rat barrel cortex. J. Comp. Neurol. 412: 83–94. 1999.

Konoike H, Iwaoki H, Miwa M, Sakata H, Itoh K, Nakamura K. Comparison of non-invasive, scalp-recorded auditory steady-state responses in humans, rhesus monkeys, and common marmosets. Scientific Reports. 12: 9210 (2022). https://doi.org/10.1038/s41598-022-13228-8.

McQuarrie DA. *Statistical Mechanics*. Harper and Row. New York. 1973.

Robinson PA, Xiao Gao X, Han Y. Relationships between lognormal distributions of neural properties, activity, criticality, and connectivity. Biol. Cybernetics. 115: 121–130. 2021. https://doi.org/10.1007/s00422-021-00871-z.

Schwenk JCB, Hagan MA, Cloherty AL, Zavitz E, Morris AP, Price NSC, Rosa MGP, Bremmer F. Neural responses to broadband visual flicker in marmoset primary visual cortex. Preprint.

van Helden HPM, Vanwersch RAP, Kuijpers WC, Trap HC, Philippens IHC, Benschop, HP. Low levels of sarin affect the eeg in marmoset monkeys: a pilot study. J. Appl. Toxicology. 2004. https://doi.org/10.1002/jat.1001.

3 Supplementary Figures and Tables

3.1 Supplementary Figures

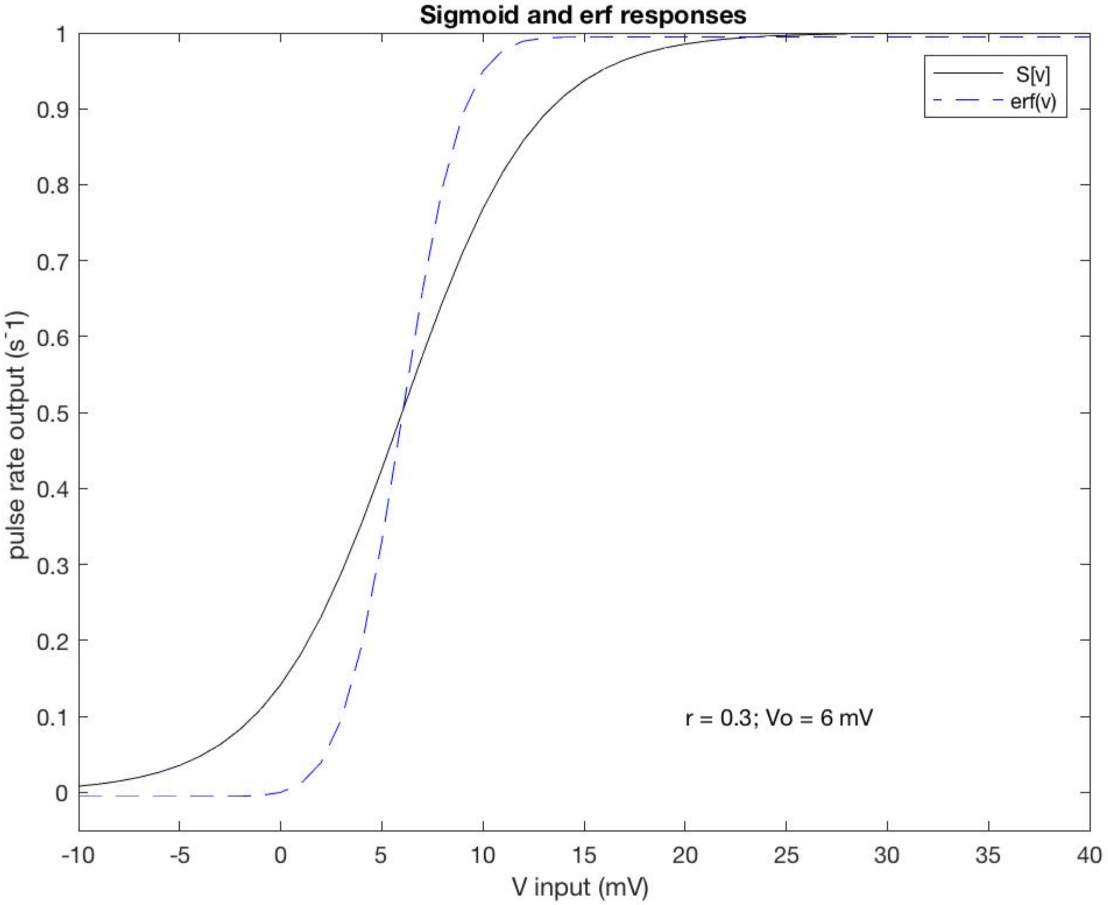

Supplementary Figure S1: Comparison of the sigmoid approximation (eq. S2) to the error function form (eq. S1) of the voltage to rate transformation function.

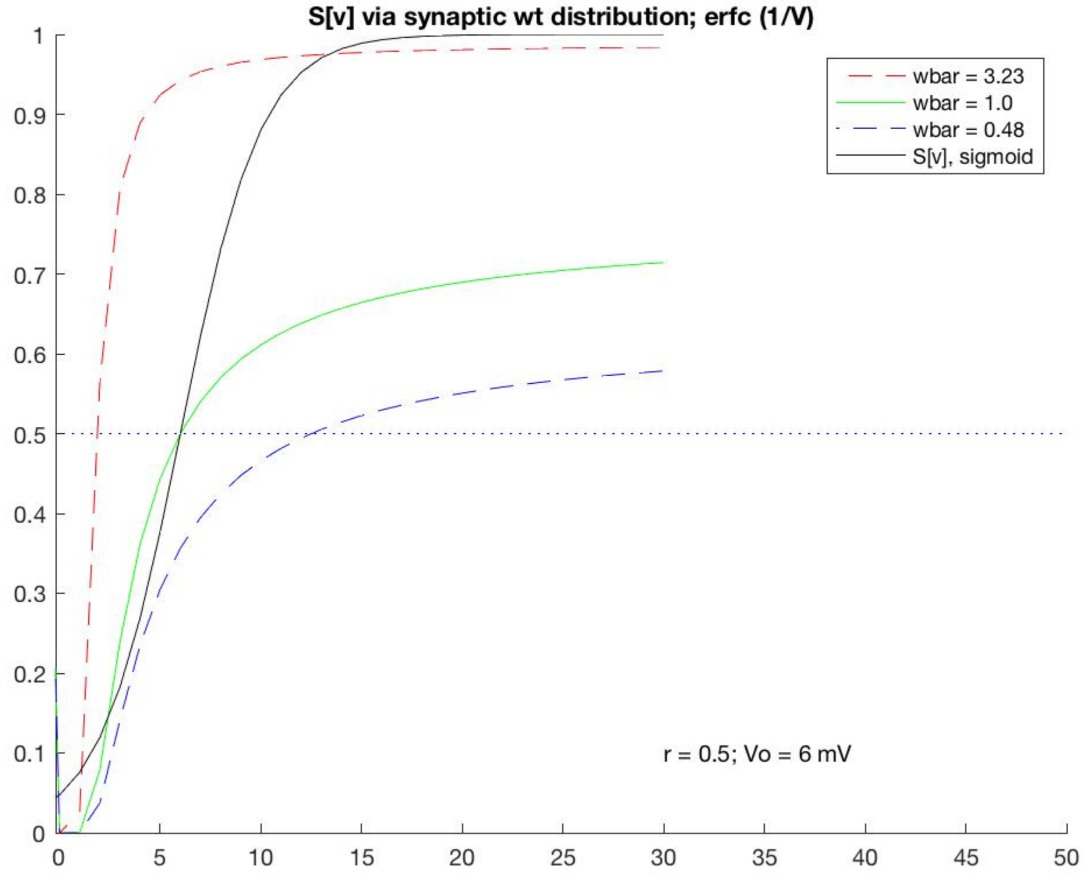

Supplementary Figure S2: Comparison of the error function form (eq. S4) of the voltage to rate transformation function to the standard sigmoid approximation (eq. S2). Higher synaptic weights shifts the curve left to lower voltage, with lower asymptotic limits at higher voltages (right).

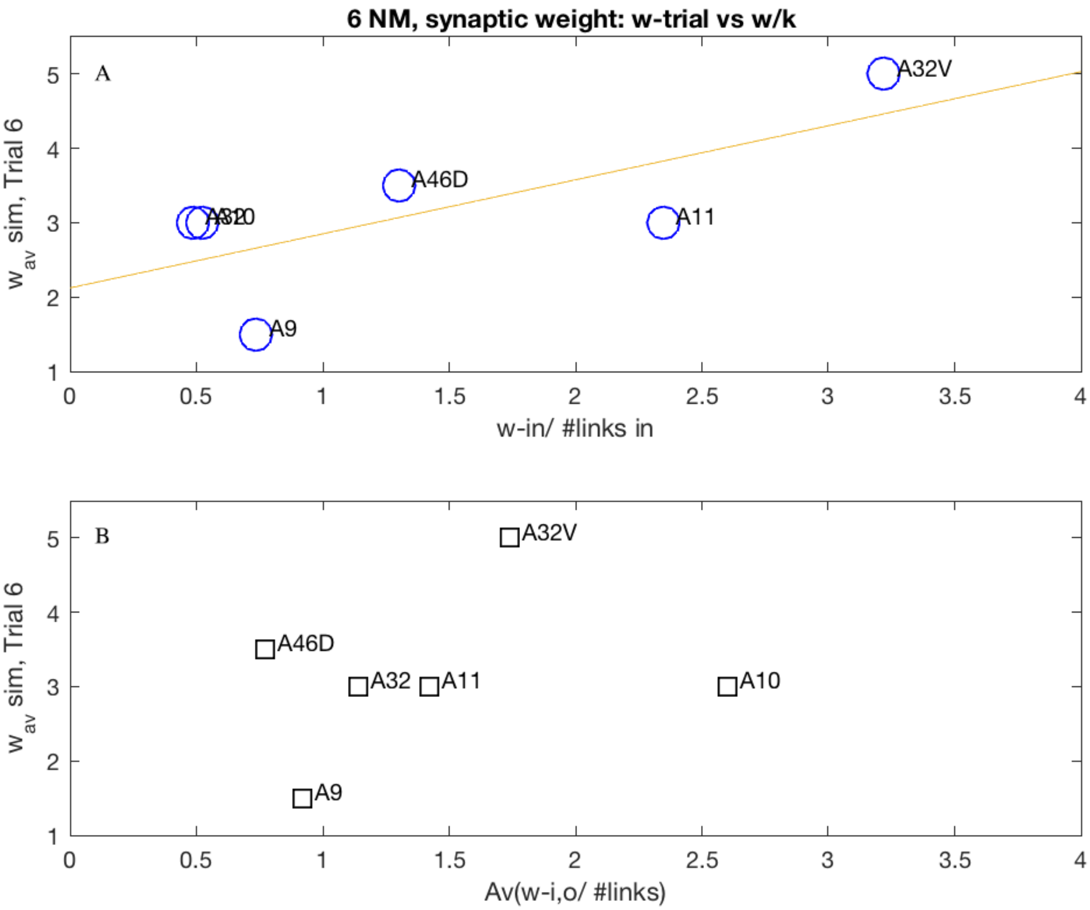

Supplementary Figure S3. Comparison of trial w_{av} with weight per in link and linear fit (A. top); and average of w/k for in and out links (B. bottom) data for marmoset (cf. Table S1).

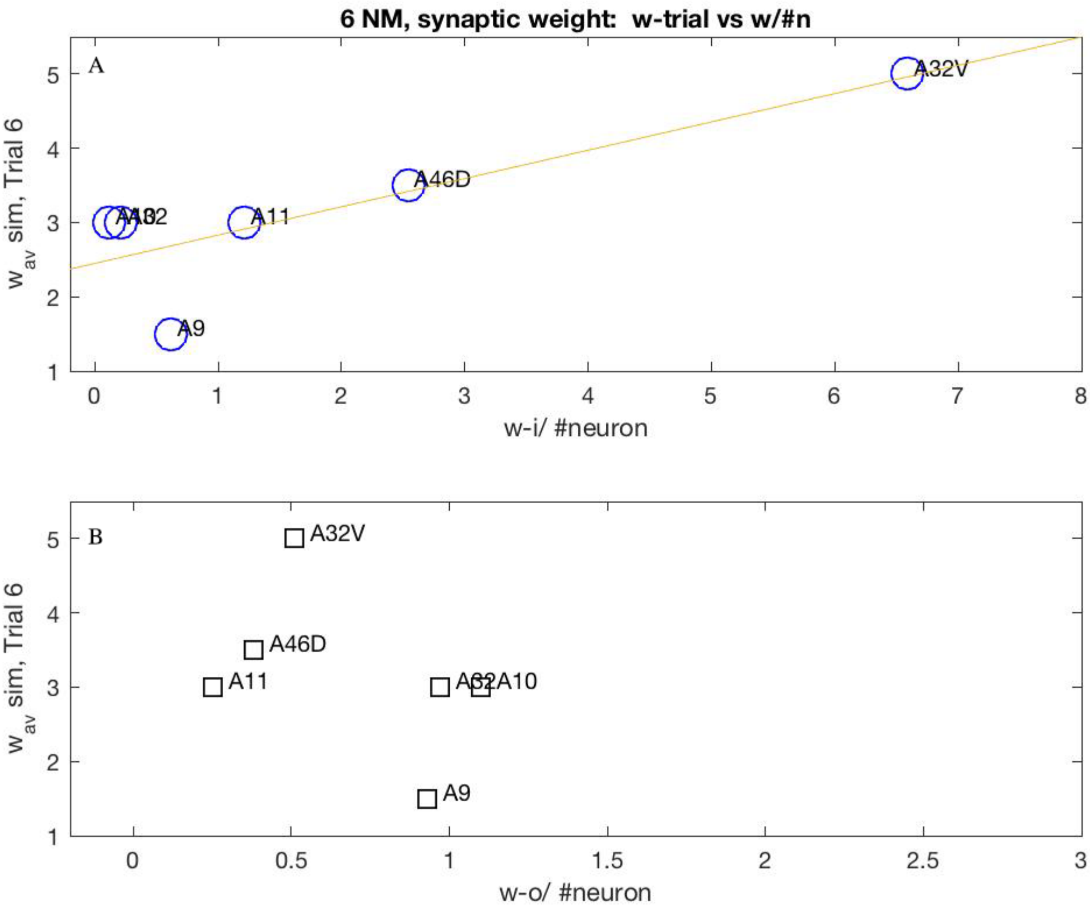

Supplementary Figure S4. Comparison of trial w_{av} vs. in weight per neuron (A. top) and out weight per neuron (B. bottom) data for marmoset (cf. Table S2).

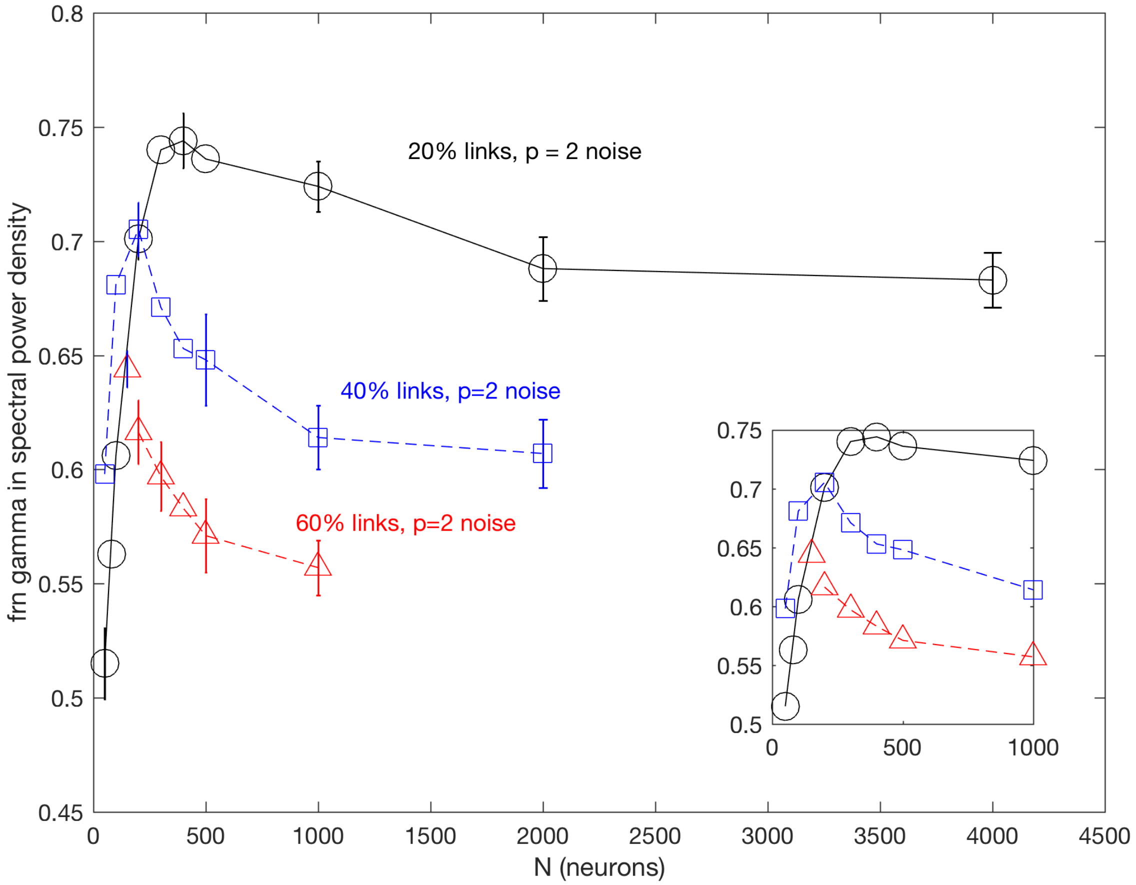

Supplementary Figure S5. Fraction of gamma (40-100 Hz) power in the total spectrum of LFP output for a simulation of N LIF neurons. Insert: detail of results for small N.

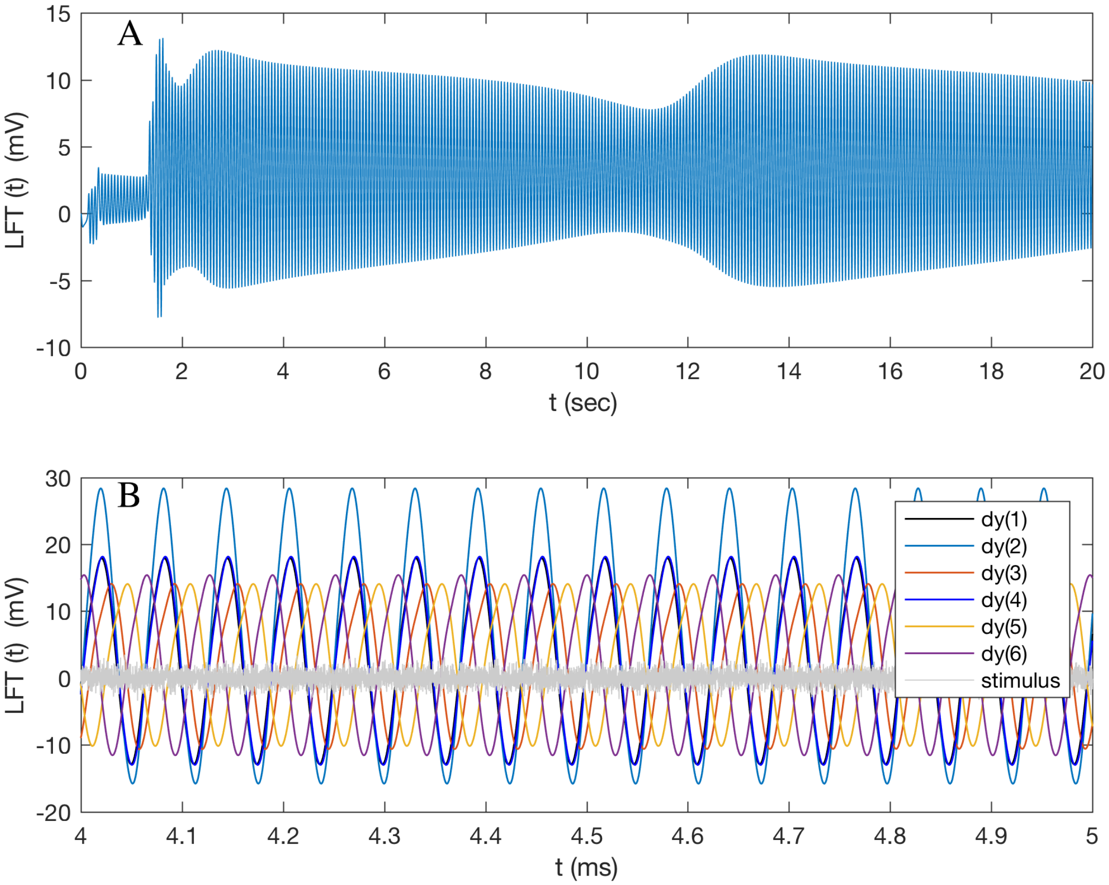

Supplementary Figure S6. LFP output from the classic sigmoid model A. c(f eq. S2), tuned in the beta band, using a sigmoid S[v] (eq. S2) and with only Gaussian random noise (sd =1) as a driving input. Output dy(t) of each node B. using a base level model, along with the noise input (grey).

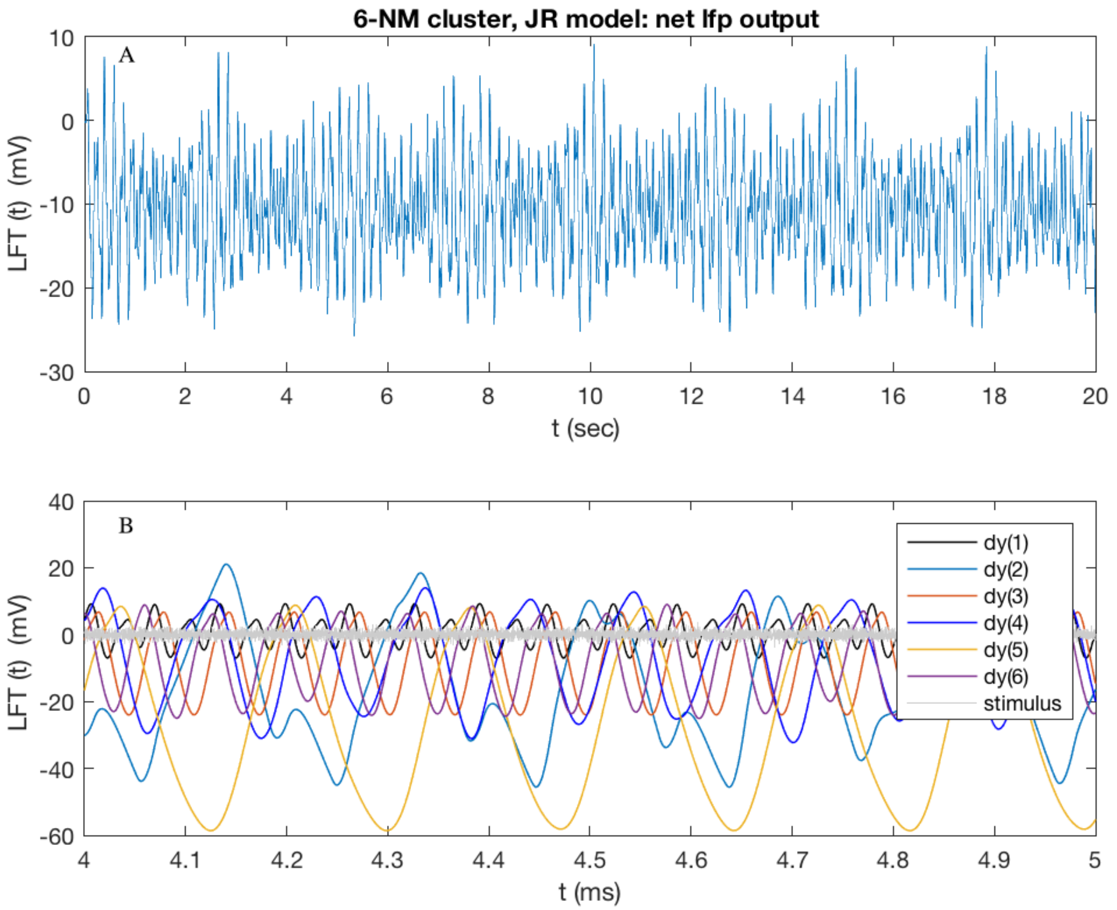

Supplementary Figure S7. Output LFP (A.) and component dy(t) of each node (B,), along with the noise input (grey), for the full model (eq. S1, 6), driven by unit sd Gaussian noise input.

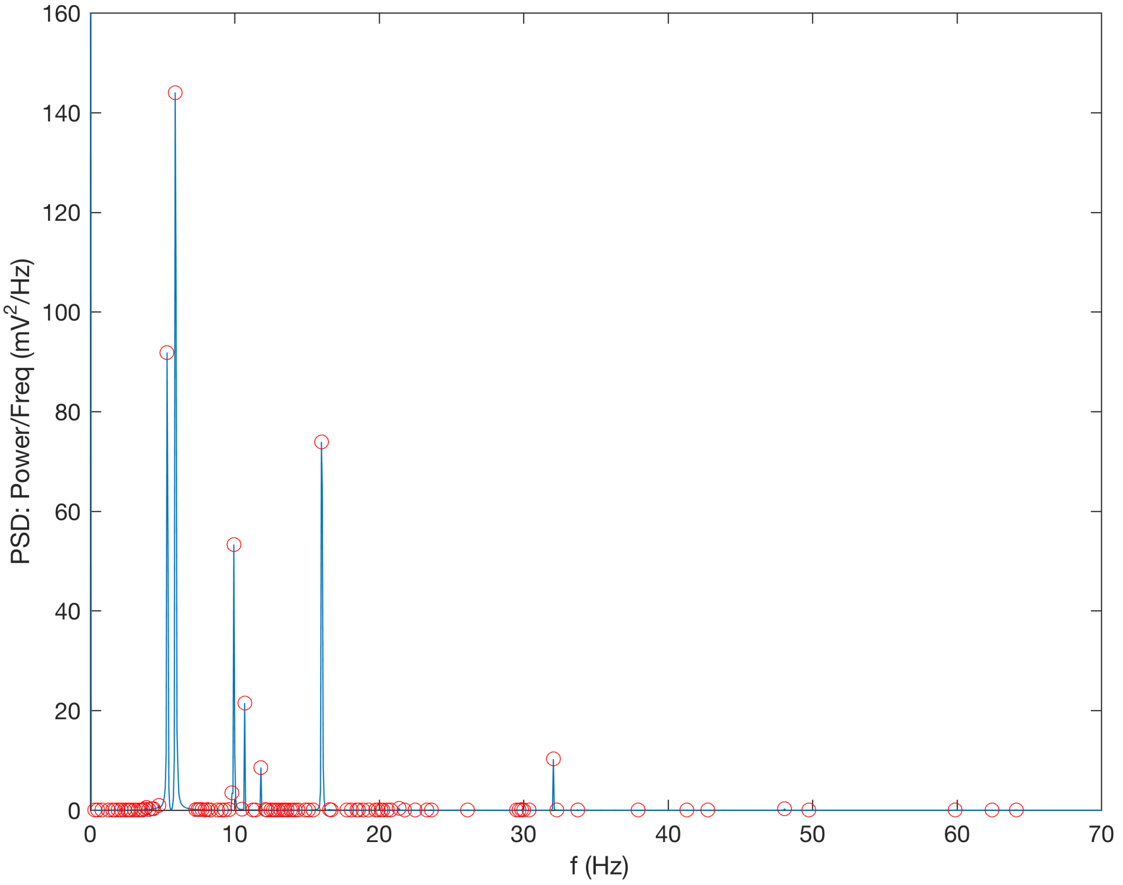

Supplementary Figure S8. Power spectrum density of resting state LFP of the six node cluster, calculated by Fourier transform with peaks highlighted (Matlab *fft*, *findpeaks*). Peaks are at 5.3, 5.9, 9.9, 10.7, 16.0 and 32.1 Hz, corresponding to the resonances (cf. Table 1) of the six nodes in the pFC cluster.

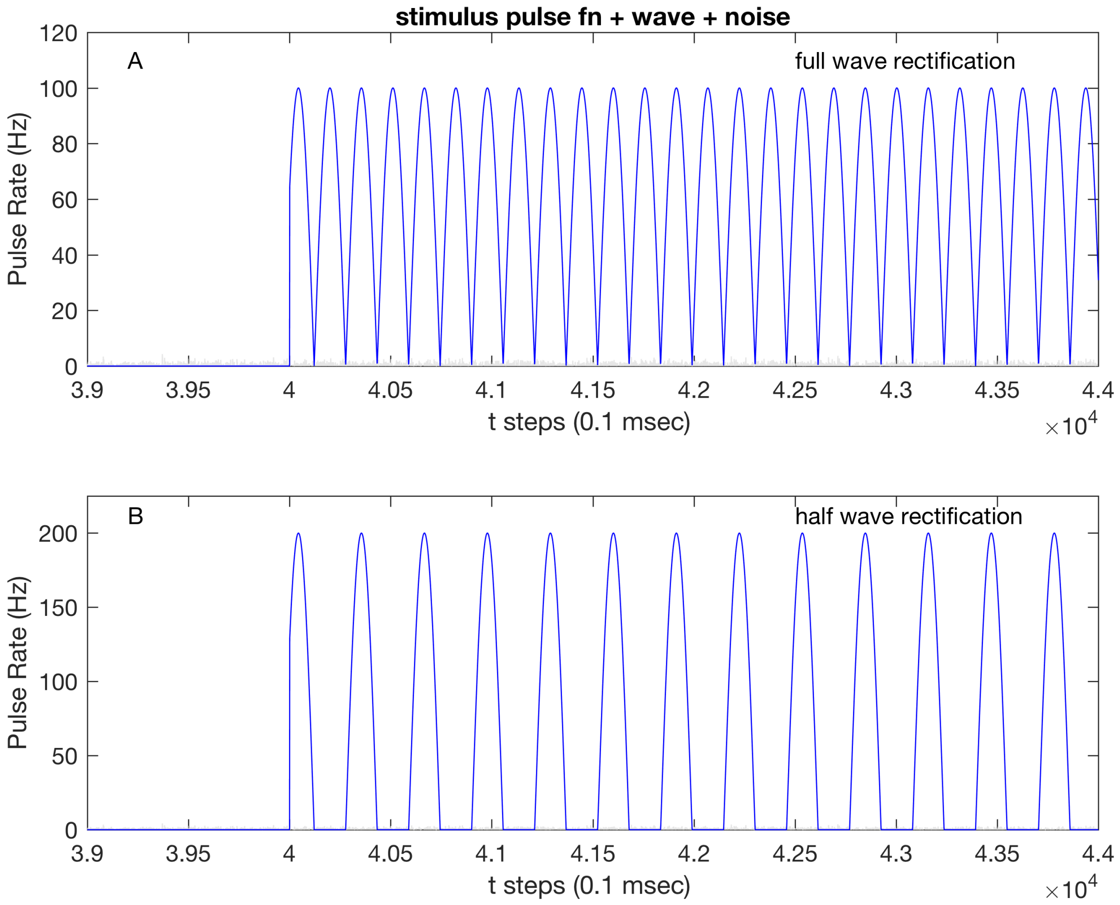

Supplementary Figure S9. A constant pulse rate, here modulated in the gamma band, used as a wave like stimulus to nodes in the cluster. Waveform is passed in full, (A) with negative amplitudes rectified to positive); or with negative phases omitted (B).

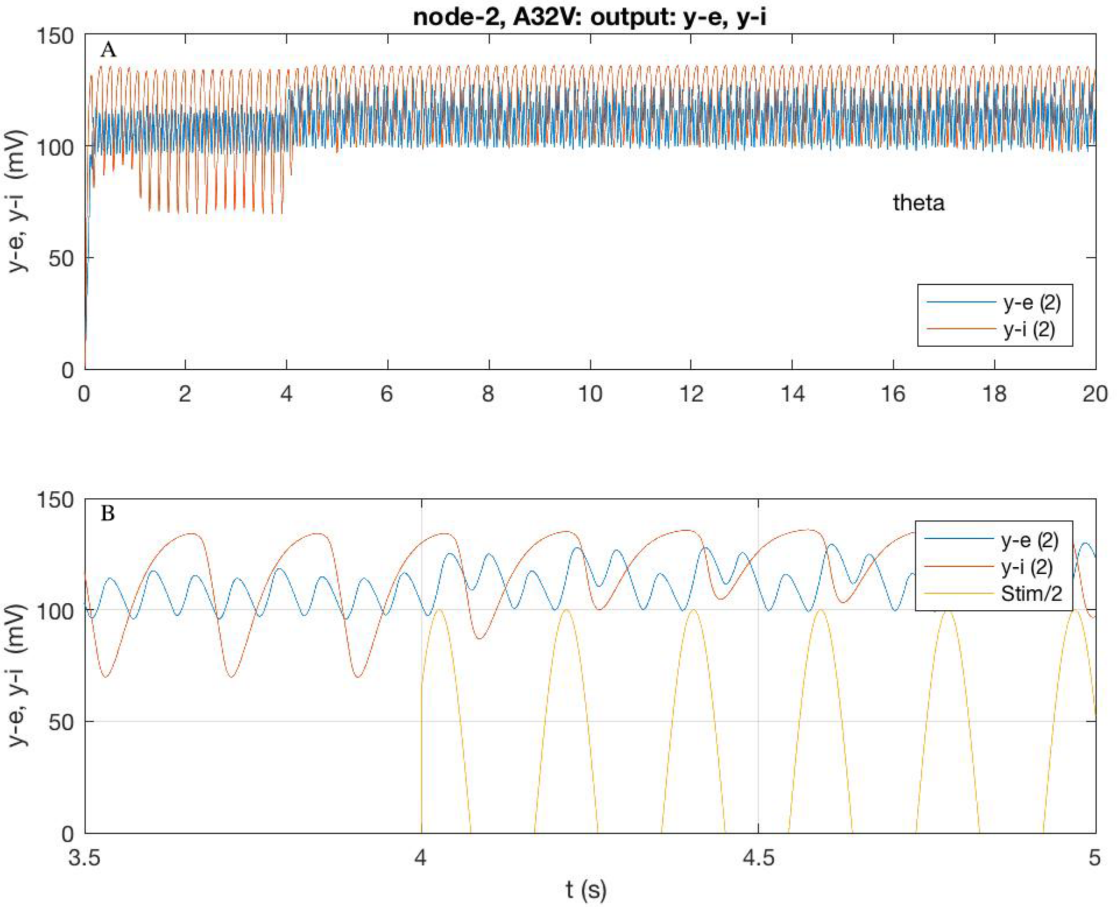

Supplementary Figure S10. Output (A) of node 2 (A32V) in response to a gamma (half wave) modulated stimulus applied to all nodes. The response is dominated by the inhibitory sub population oscillation (B).

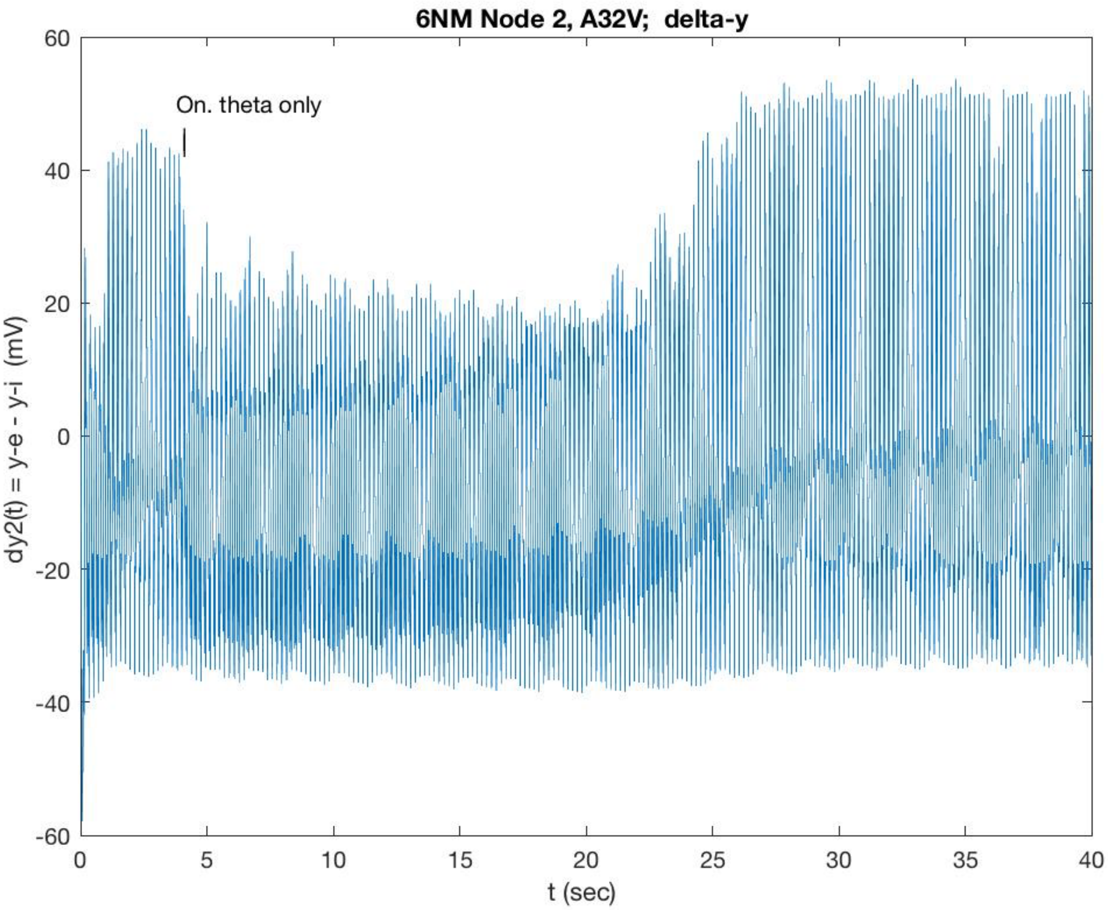

Supplementary Figure S11. Response of node A32V to a theta modulated stimulus to nodes A32V and A11 applied at 4 s. This shows a long lasting theta induced transition that relaxes back to a high amplitude state after 17 s.

3.2 Supplementary Tables

#	Acrn	Vol (mm^3)	h (mm)	N (x 10^5)	k_{in}	w_{in} (x 10^3)	k_{out}	w_{out} (x 10^3)	Ratio w/k	Rescaled w_{av}
1	A10	23.9	1.9	21.32	5	2.61	5	23.42	2.60	3.38
2	A32V	2.7	1.0	2.44	5	16.1	5	1.25	1.74	2.25
3	A32	11.4	1.4	9.24	4	1.94	5	9.0	1.14	1.48
4	A9	7.7	1.8	5.97	5	3.68	5	5.55	0.92	1.2
5	A46D	1.2	1.65	2.56	5	6.5	4	0.96	0.77	1.0
6	A11	12.0	1.35	9.73	5	11.73	5	2.42	1.42	1.84

Supplementary Table S1. Information (cf. text) on the anatomical areas in the 6-node cluster. Number, k, and total weight, w, are for links internal to the cluster.

#	Acrn	N (x 10^5)	w_{in} (x 10^3)	w_{out} (x 10^3)	w_{in} /N (x 10^2)	w_{out} /N (x 10^2)
1	A10	21.32	2.61	23.42	0.12	1.1
2	A32V	2.44	16.1	1.25	6.59	0.51
3	A32	9.24	1.94	9.0	0.21	0.97
4	A9	5.97	3.68	5.55	0.62	0.93
5	A46D	2.56	6.5	0.96	2.54	0.38
6	A11	9.73	11.73	2.42	1.21	0.25

Supplementary Table S2. Weight per neuron of local in and out links in the cluster derived from marmoset connectivity data.

#	Acrn	k_{in}	w_{in} (x 10^3)	k_{out}	w_{out} (x 10^3)
1	A10	87	4.14	25	3.87
2	A32V	46	17.0	7	0.032
3	A32	32	2.73	17	2.18
4	A9	54	2.66	20	2.24
5	A46D	58	8.68	21	0.84
6	A11	76	10.9	26	1.5

Supplementary Table S3. Additional information on the anatomical areas in the 6-node cluster. Number of links, k and sub total of link weights, w (in, out) are for external links only – ie. with areas outside the 6-node cluster - the internal links listed in Table S1 are not counted here.

Frequency band	C	r	w_{av}	τ_e (ms)	τ_ι (ms)	f (Hz)
theta	180	0.5	1.5	20	22	6.1
alpha	200	0.43	1.5	15	16	9.1
beta	250	0.5	3	10	10	15.4
gamma	350	0.56	5	5	5	32.1

Supplementary Table S4. Typical parameter values (cf. eq. 3 - 6) for a single node, to produce oscillations in the standard frequency bands. f is the dominant spectral peak found in the exploratory simulation.

band	C	r	w_{av}	t_e (ms)	t_i (ms)	dy (mV)	f (Hz)
theta	180	0.4	2.0	20	22	0.1	no osc.
		0.5				45.2	5.5
		0.56				46.1	6.5
	180	0.5	1.5	20	22	41	6.1
		0.5	2.0			45.2	5.5
		0.5	3.0			48.4	4.3
alpha	200	0.4	2.0	15	16	28.7	8.0
		0.43				30.3	8.5
		0.5				33.5	9.3
		0.56				35.9	9.83
beta	250	0.4	1.0	10	10	11.5	15.83
		0.4	1.5			17.6	15.7
	250	0.5	2.0			27.0	15.8
		0.5	3.0			30.1	15.4
	250	0.56	1.0			0.1	no osc.
			2.0			29.4	15.83
			3.0			32.1	15.5
gamma	350	0.5	5.0	5	5	21.7	30.7
		0.56	5.0			23.0	30.8

Supplementary Table S5. A range of parameters, in eq. 3-6, output potential (dy $=y_e - y_i$, max peak-to-peak) and main spectral peak. Parameters are varied one at a time.

Node	Frequency band	C	r	w_{av}	τ_e (ms)	τ_i (ms)	dy (mV)	f (Hz)
A11	theta	180	0.5	1.5	20	22	41	6
A46D	alpha	200	0.43	1.5	15	16	25.6	9.2
A9	beta	250	0.4	3	10	10	30.1	15.5
A10	gamma	350	0.5	5	5	5	21.7	30.7

Supplementary Table S6. Typical parameter values (cf. eq. 3-6) assigned to the 4 node pre cluster, and resultant frequency bands. Resultant peak-to-peak amplitude of the output dy is listed; f is the dominant spectral peak frequency.

Acrn	Vol (mm^3)	neuron density ($10^3/mm^3$)	h (mm)	N (x 10^5)	Surface Area (mm^2)	No. Colm.	No. Mini Colm.	neurons / Mini-Colm
A10	23.9	89.1	1.9	21.32	12.59	177	5037	423
A32V	2.7	91.3	1.0	2.44	2.68	38	1071	228
A32	11.4	80.8	1.4	9.24	8.17	115	3267	283
A9	7.7	77.2	1.8	5.97	4.30	61	1720	347
A46D	1.2	77.8	1.65	2.56	1.99	28	797	321
A11	12.0	80.85	1.35	9.73	8.91	126	3565	273

Supplementary Table S7. Geometrical characteristics (cf. text) of the anatomical areas in the 6-node cluster. The right most column lists the estimated number of neurons per mini-column.
